# Cocrystals and Solvates are Not the Same: A Network Perspective

**DOI:** 10.1002/cphc.202500338

**Published:** 2025-10-01

**Authors:** Tom Edward de Vries, Hugo Meekes, Elias Vlieg, René de Gelder

**Affiliations:** ^1^ Radboud University Solid State Chemistry Institute for Molecules and Materials Heyendaalseweg 135 6525AJ Nijmegen The Netherlands

**Keywords:** Cocrystals, Crystal engineering, Network analysis, Solid‐state structures, Solvates

## Abstract

Cocrystals and solvates are two kinds of multicomponent crystals. Cocrystals consist of solid compounds, while solvates consist of a mixture of solid and liquid compounds. This work addresses the question of whether cocrystals and solvates can be treated the same or need to be treated separately. To do this, networks out of known cocrystals and solvates are created, where each node represents an individual compound, and two nodes are linked if the compounds they represent form a cocrystal or solvate. These networks are also used to predict new cocrystals and solvates using a technique called link prediction. By analyzing the structure of the cocrystal and solvate networks, differences in behaviors between cocrystals and solvates are found, due in part to a clash between chemical and steric complementarity found in the solvate network. Based on the analysis of the networks, it is found that cocrystals and solvates cannot be treated equally and that information from one network is not relevant for predictions in the other. It is also found that link prediction performs poorly when used on the solvate network, unless 14 problematic solvents are removed.

## Graphical abstract

Based on network analysis, cocrystals and solvates cannot be treated the same.

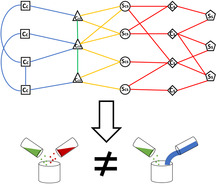

## 1 Introduction

Multicomponent crystals (MCCs) are crystals composed of at least two different molecular or ionic components. MCCs have gained a lot of interest, particularly due to their applications in the pharmaceutical industry. If an active pharmaceutical ingredient (API) has a property that is counteractive to its intended use, it may be possible to form an MCC with another compound. In general, this MCC will have properties different from the compounds used to create it. By finding the right cocrystallizing compound, it may be possible to design a new API form that has the desired properties.^[^ ^1^, ^2^, ^3^, ^4^ ^]^

MCCs can be divided into three main categories: (1) cocrystals, which consist of two or more solid neutral components called coformers; (2) solvates, which consist of at least one coformer and one solvent; and (3) salts, which consist of charged components. When three or more components are involved, there are more possibilities: cocrystal solvates, cocrystal salts, salt solvates, and cocrystal salt solvates.^[^ ^5^ ^]^ Although it has been shown that this division is not always clear cut,^[^ ^6^ ^]^ these categories are a convenient way of classifying MCCs.

Naively, one might state that the only difference between a cocrystal and a solvate is that one of the components of the solvate has a melting point below room temperature. However, there are other differences between the two types of MCCs. Molecular size is one such difference: most solvents are small compared to the other components. Another difference is that cocrystal synthesis often involves a three‐component system (two coformers and a solvent), while solvate synthesis usually involves a two‐component system (a coformer and a solvent). There are also physicochemical differences. For instance, cocrystals tend to have a single melting point that can be observed using differential scanning calorimetry (DSC). Solvates, on the other hand, often show additional endothermic peaks in the low‐temperature regime of the DSC curves related to the loss of solvent. This difference can be used to determine whether a given MCC is a solvate or a cocrystal, but examples have been found of cocrystals that show the additional DSC peaks expected for solvates.^[^ ^7^ ^]^ This raises the question of whether the distinction between cocrystals and solvates is artificial. In this article, we aim to find out if solvates and cocrystals can be treated as equals or if they need to be treated separately when discussing MCCs.

In previous work, a method for predicting MCCs called link prediction was investigated.^[^ ^8^, ^9^, ^10^ ^]^ A network was created out of known cocrystals extracted from the Cambridge Structural Database (CSD). In this cocrystal network, individual coformers are represented by the nodes, and cocrystals are represented by the links between the nodes. New links (and thus new cocrystals) could be predicted in this network using network science techniques. This link prediction technique does not use explicit chemical information, instead relying solely on the structure of the cocrystal network. At the time, this network was limited to cocrystals and coformers only. By creating another network out of all known solvates, we here compare the cocrystal and solvate networks to find similarities and differences between cocrystals and solvates. By looking at various network properties, we are able to examine these differences for far greater numbers than if we were to look at (chemical) properties of individual solvates and cocrystals.

The cocrystal network was found to have a strong bipartite structure owing to the chemical complementarity of coformers that successfully cocrystallize.^[^ ^11^ ^]^ This complementarity often stems from coformers forming a donor‐acceptor pair. Solvent molecules are known to take on various roles in solvate formation: hydrogen bond donor/acceptor, filling empty space, ligands, and polar–apolar bridges.^[^ ^12^ ^]^ We will examine whether despite this, the solvate network has a bipartite structure similar to that of the cocrystal network. This will tell us whether solvates follow a similar pattern of chemical complementarity.

It has also been reported that the cocrystal network has a scale‐free structure.^[^ ^13^ ^]^ We will analyze the solvate network to find out whether it has the same scale‐free structure.

Solvate prediction has been done using machine learning,^[^ ^14^, ^15^ ^]^ but network‐based link prediction has not been attempted yet. Whether or not link prediction can work for solvates will heavily depend on the structure and information content of the solvate network. Finally, in order to find out if cocrystals and solvates can be treated the same, we will combine the cocrystal and solvate networks to see if this improves the accuracy of link prediction algorithms. This will tell us whether information on solvate formation is relevant to predictions on cocrystal formation and vice versa.

## 2 Methodology

### 2.1 Network Construction

Our networks are created using the CSD (version 5.44, including February 2024 update). The CSD python API^[^ ^16^ ^]^ (Application Programming Interface, not to be confused with active pharmaceutical ingredient) is used to extract all CSD entries that are organic, have no errors, are not polymeric or ionic, contain exactly two different residue types, and have their 3D coordinates determined. The list of entries is then separated into solvates and cocrystals using a custom classifier algorithm written by Devogelaer et al.^[^ ^13^ ^]^ A unique canonical SMILES code is generated for each MCC. In canonical SMILES codes, different molecular components are separated by a “.” character, making it easy to split the MCC into its constituent components. Two networks are created, one for solvates and one for cocrystals. In each network, a node is created for every compound. The nodes are marked as either coformer (for solid components) or solvent (for liquids). The distinction is made using a list of canonical SMILES codes of known solvents that appear in the CSD (see SI). For every solvate or cocrystal, a link is created between the nodes representing its two constituent components. The networks are stored in an *N* × *N* adjacency matrix *A*, where *N* is the number of nodes and *A*_*i,j*_ = 1 if there is a link between nodes *i* and *j*, and *A*_*i,j*_ = 0 otherwise.

**Figure 1:**
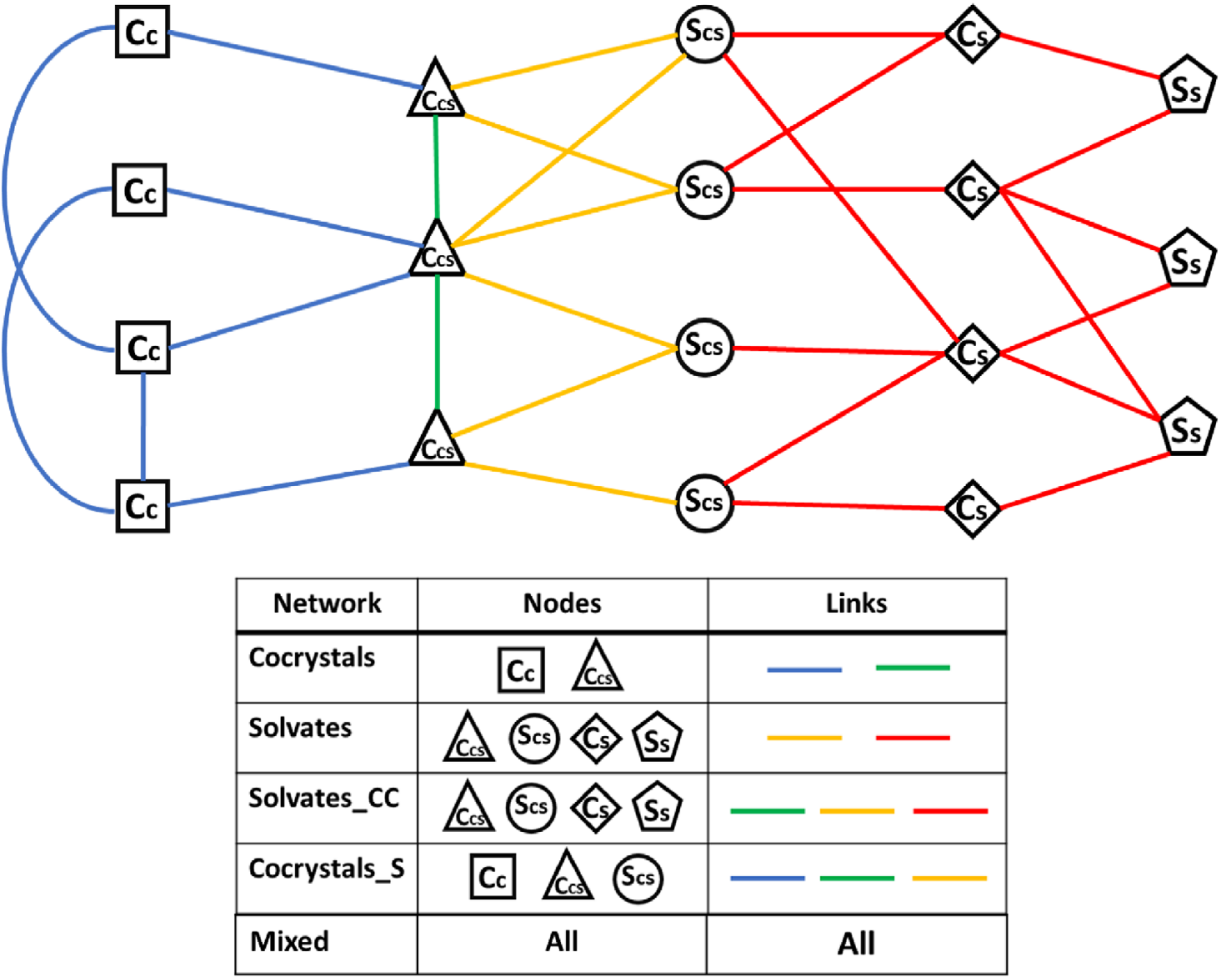
An example network for demonstrative purposes. Nodes marked with **C** represent coformers; nodes marked with **S** represent solvents. Blue and green links represent cocrystals; red and yellow links represent solvates.

In some cases, it is interesting to mix certain parts of the two networks. To explain the various networks, we refer to the example network in **Figure** **1**. We distinguish three different types of coformer nodes and two kinds of solvent nodes. Starting with the coformers: squares marked **Cc** are coformers that only form cocrystals, triangles marked **Ccs** are coformers that form both cocrystals and solvates, and diamonds marked **Cs** are coformers that only form solvates. For the solvents: circles marked **Scs** are solvents that connect to at least one coformer in the cocrystal network (so at least one triangle), and pentagons marked **Ss** are solvents that do not connect to any coformers in the cocrystal network.

We will make use of five different networks. The first is the cocrystal network, which contains all coformers that can form at least one cocrystal (squares and triangles in Figure 1), and only contains the cocrystal links between these coformers (blue and green).

Second is the solvate network, which contains all coformers (triangles and diamonds) and solvents (circles and pentagons) that form at least one solvate, and only contains the solvate links between them (red and yellow).

Third is the Solvates_CC network, which is created by taking the solvate network and adding cocrystal links between coformer nodes that were already in the solvate network (green).

Fourth is the cocrystals_S network, which is created by taking the cocrystal network and adding only those solvents that form solvates with coformers that were already in the cocrystal network (yellow links and circular nodes).

Finally, we have the mixed network, which contains all coformers and solvents, and all cocrystal and solvate links between them.

### 2.2 Link Prediction

Link prediction algorithms are designed to predict the next link(s) to appear in an evolving network. They work by giving a score to each pair of unlinked nodes in the network.

There are many different scoring functions, and the scoring function called multistep resource allocation (MSRA) was previously found to be the most accurate for the cocrystal network. The MSRA score is based on counting paths of various lengths between two target nodes. It was found that counting three‐step paths (MSRA3) resulted in the most accurate predictions.^[^ ^10^ ^]^ For this work, we also tested MSRA3 and various other scoring functions on the solvate network and found that MSRA3 is still the best method (see SI). To calculate the MSRA3 score for a pair of nodes (*i*, *j*), the algorithm first identifies the neighbors of *i* and *j*. It then checks if any of the neighbors of *i* link to the neighbors of *j*. If so, there is a path with a length of three steps from *i* to *j*. Each of these paths *i − n*_*i*_ *− n*_*j*_ *− j* is then given a score of $\frac{1}{d(n_{\text{i}})d(n_{\text{j}})}$ where *d*(*n*) is the degree of node. The scores for all three‐step paths are added together to obtain the final score for the link (*i*, *j*).

## 3 Results and Discussion

### 3.1 Network Construction

**Figure 2:**
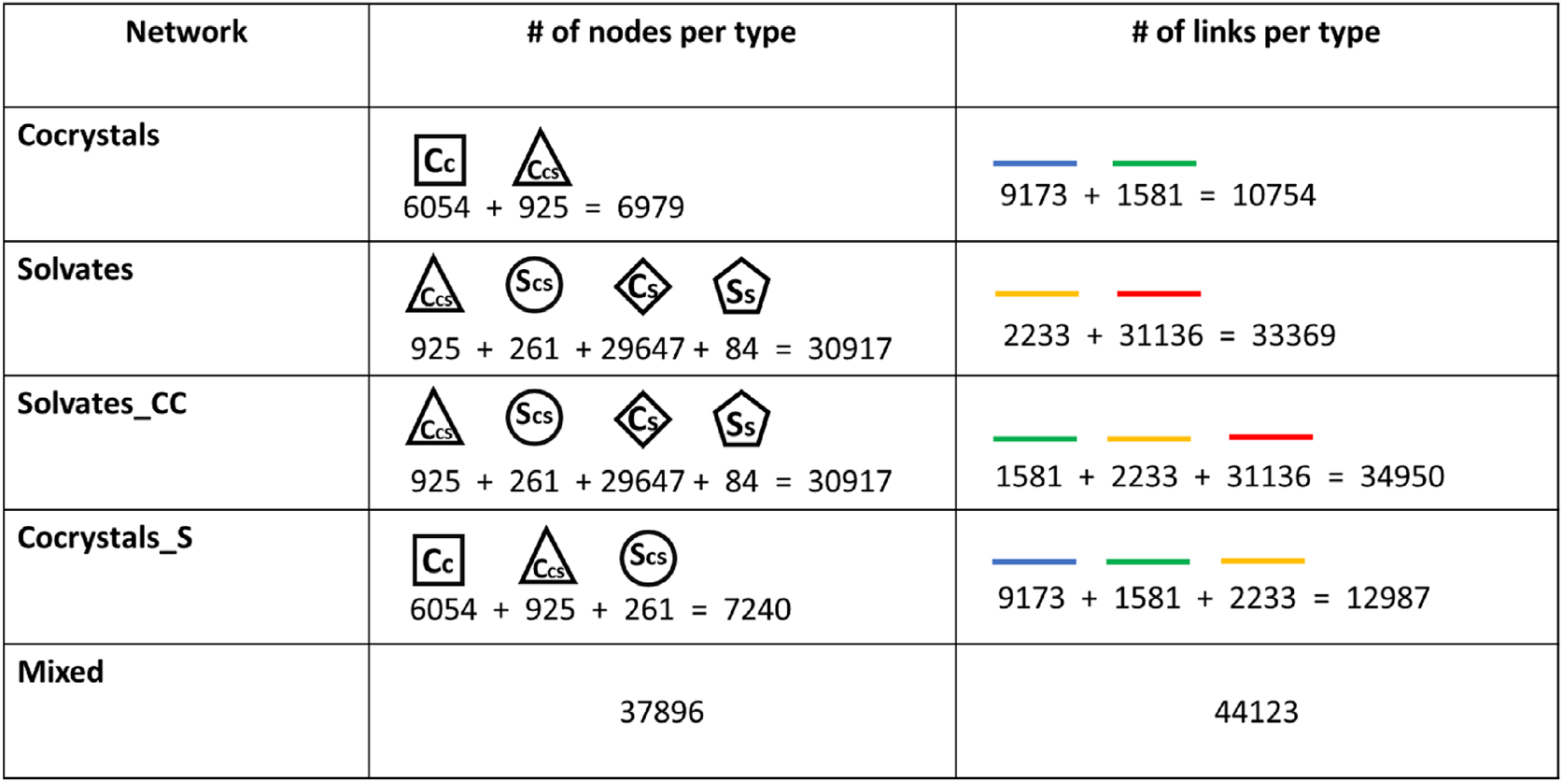
The number of nodes and links of every type in each network.

In this section, we will examine the five networks by looking at the numbers of different nodes and links. The numbers of nodes and links of every type in each network are shown **Figure** **2**.

It is clear that the cocrystal and solvate networks are quite different. The solvate network is about three times as large (in terms of links) as the cocrystal network, and it contains a lot more coformers than solvents. This large imbalance means that every coformer node in the solvate network must connect to a very small group of solvate nodes. This kind of structure is not seen in the cocrystal network. Both the size and imbalance of the solvate network can be explained by serendipitous solvate formation during crystallization of compounds from solution, as well as the limited number of available solvents. We can also see that the link density in the cocrystal network is higher than that of the solvate network. Nodes in the cocrystal network have 1.54 links on average, while nodes in the solvate network have 1.08 links on average.

The overlap between the cocrystal and solvate network consists of 925 nodes connected to each other by 1581 links. This overlap is only a fraction of the two networks (13% of the cocrystal network and 3% of the solvate network). The fact that the overlap is so small may suggest that, in the mixed network, the cocrystal and solvate parts behave as isolated subgraphs. However, with 1.71 green links per node on average (see Figure 1), the overlap is a relatively well‐connected part of both networks. This means that the overlap could still have a significant impact on predictions made in networks containing both solvates and cocrystals. There are only 84 solvents that have no links to any of the overlapping coformers, these solvents are rather exotic and not used often.

### 3.2 Degree Distribution and k‐core Analysis

**Figure 3:**
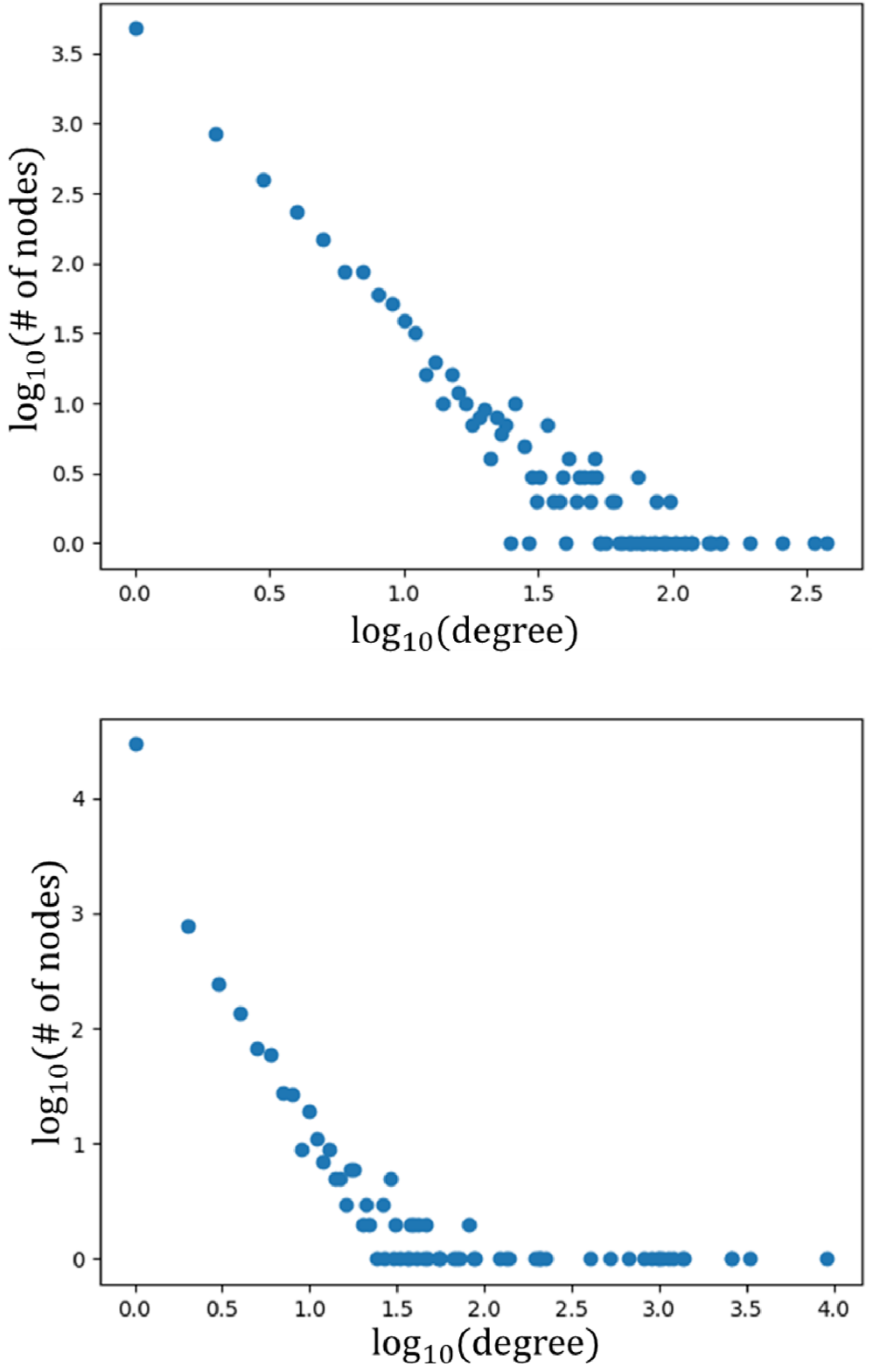
Degree distributions for the cocrystal (top) and solvate (bottom) networks on a log‐log scale.

The degree distribution of the cocrystal network was first examined by Devogelaer et al. who discovered that the degree distribution matches a power law model, recognizable by the linear shape in a log‐log scale, which means that the network is considered scale‐free.^[^ ^13^ ^]^ We have recreated this degree distribution for comparison with that for the solvate network (see **Figure** **3**). Both degree distributions show linear behavior at lower degrees that “fans out” at higher degrees. One difference between the two is that the solvate network has more nodes above log *d* = 2.0. Due to this, the degree distribution of the solvate network has a clear “kink” around log *d* = 1.5, which suggests the network is only partly scale free.

**Figure 4:**
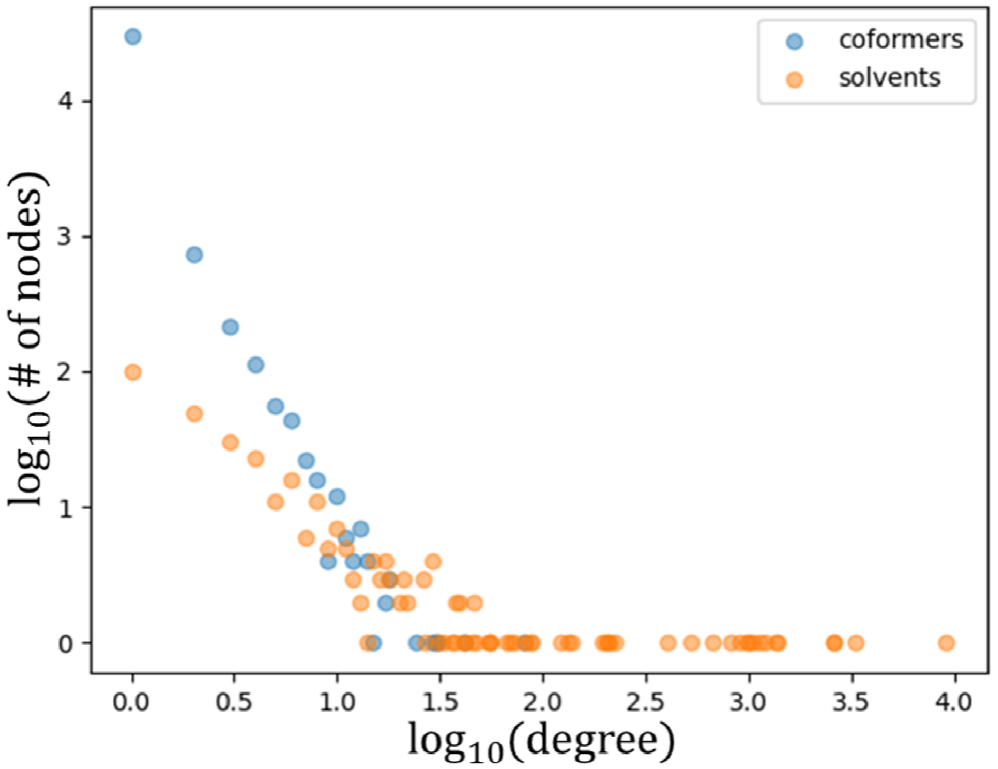
Separate degree distributions for the solvents and coformers in the solvate network.

The main difference between solvates and cocrystals is of course that solvates contain both a solvent and a coformer, while cocrystals contain only coformers. To see if there is a difference in node properties between solvents and coformers, we can split the degree distribution for the solvate network into two, one for the solvents and one for the coformers. These distributions are shown in **Figure** **4**.

The distribution for coformers in the solvate network is similar to the cocrystal distribution in Figure 3. The large number of points at the high degree end of Figure 4 belong to solvents. The linear part of the solvent degree distribution has a different slope from the one observed for the coformers and for the cocrystal network. This implies that the solvents do not follow the same power law as the coformers, if they follow one at all. We will examine whether this structural difference has implications for other network properties and if we might be able to identify whether certain nodes are responsible for this different behavior.

**Figure 5:**
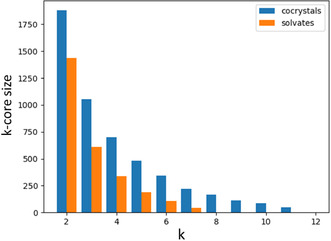
The number of nodes in each maximal k‐core for the cocrystal and solvate networks.

To get a more specific idea of how well‐connected the networks are, we can plot the sizes of all maximal k‐cores in both networks. A maximal k‐core is a subset of a network where every node has at least k links (see SI). To do this, we simply find the maximal k‐core for increasing values of *k* in both networks until they are both empty. **Figure** **5** shows the sizes (in # of nodes) of all maximal k‐cores in the cocrystal and solvate networks. The 1‐core is not shown, as this is simply the entire network.

Even though the solvate network is bigger, every maximal k‐core in the solvate network is smaller than the equivalent k‐core in the cocrystal network. In fact, the 2‐core of the solvate network consists of less than 5% of the full network. On top of that, the solvate network has no k‐cores beyond *k* = 7, while the cocrystal network has k‐cores up to *k* = 11. This shows us that the cocrystal network is much more interconnected than the solvate network, despite the latter containing more nodes and links.

To show that the k‐core distributions are significantly different from those of random networks, we also compared the k‐core distributions of the cocrystal and solvate networks to the average distributions of 100 randomly generated networks with the same number of nodes and links as the cocrystal and solvate networks (see SI).

### 3.3 Bipartization

#### 3.3.1 Bipartizing the Solvate Network

The cocrystal network has previously been shown to be nearly bipartite. A network is considered bipartite if its nodes can be split into two groups *A* and *B* such that there are no links from nodes in group *A* to other nodes in group *A* and no links from nodes in group *B* to other nodes in group *B*.

The solvate network is an inherently bipartite network. Every link in the solvate network must contain one solvent and one coformer, so we can simply say group *A* consists of solvents and group *B* consists of coformers. However, we imposed this bipartization with our decision to only allow solvate links. By using the Solvates_CC network, we can examine the complementarity between coformers and solvents. If the cocrystal and solvate networks share a compatible bipartite structure, we expect that the Solvates_CC network is closer to being bipartite than the cocrystal network.

We have previously found that 4% of the links (≈500 links) in the cocrystal network need to be removed to bipartize the network using our algorithm.^[^ ^11^ ^]^ The solvate network is 100% bipartite by definition. Bipartizing the Solvates_CC network requires only 2% (≈700 links) to be removed. It seems like the Solvates_CC network is closer to being bipartite than the cocrystal network. However, these numbers are misleading. Both networks contain a large number of nodes with a degree of 1. These nodes do not affect to the bipartite structure of the network, but they do increase the total size of the network. Thus, to get a better sense of how close a network is to being bipartite, we can recalculate the percentages while counting only nodes with degree 2 or higher (see SI). The number of links that need to be removed has not changed, but the percentages have: 7% for the cocrystal network and 12% for the Solvates_CC network. The Solvates_CC network is actually much further from being bipartite, which means there is a clash between the bipartite structures of the cocrystal and solvate networks. We will more closely examine the solvents to identify the cause of this clash later.

**Figure 6:**
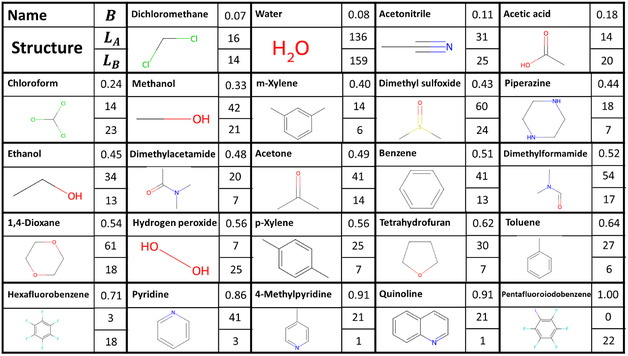
Solvents with degrees of 20 or more in order from least (top left) to most (bottom right) bipartite.

Now that we know which links are part of the bipartite network (the bipartite links) and which links need to be removed (the monopartite links), we can identify which nodes have the largest number of monopartite links. These most monopartite nodes might explain the difference in degree distribution seen in Figure 4. However, as seen in Figure 4, the unusual behavior of the solvents is at the high‐degree end. If the “unusual” nodes have a large number of links, they might influence which nodes end up in which bipartite group and thus which links are marked as monopartite. This could mean that the entire bipartization of the Solvates_CC network is incorrect. To get around this problem, we can use the bipartization of the cocrystal network as a benchmark. We know the cocrystal network shows no unusual behavior, and it is nearly bipartite. After bipartizing the cocrystal network, we can determine which coformers belong to one bipartite group (which we will call *A*) and which coformers belong to the other group (*B*). Because the cocrystal network is much closer to being bipartite, we can be reasonably certain that this is a correct and meaningful bipartization. Next, we can look at the solvate links in the cocrystals_S network. By counting how many links a solvent has to each of the two bipartite groups, we can see how well it fits into the bipartized cocrystal network. **Figure** **6** shows the solvents with a degree of 20 or higher. The solvents are ordered from lowest to highest “Bipartiteness” (*B*). We define $B=\frac{\left|L_{A}-L_{B}\right|}{\text{deg}}$ with *L*_A_ and *L*_B_ the number of links to groups *A* and *B*, respectively, and deg the degree of the node. A solvent with *B* = 1 is perfectly bipartite, and one with *B* = 0 is completely monopartite.

#### 3.3.2 Chemical Interpretation of Monopartiteness

**Figure 7:**
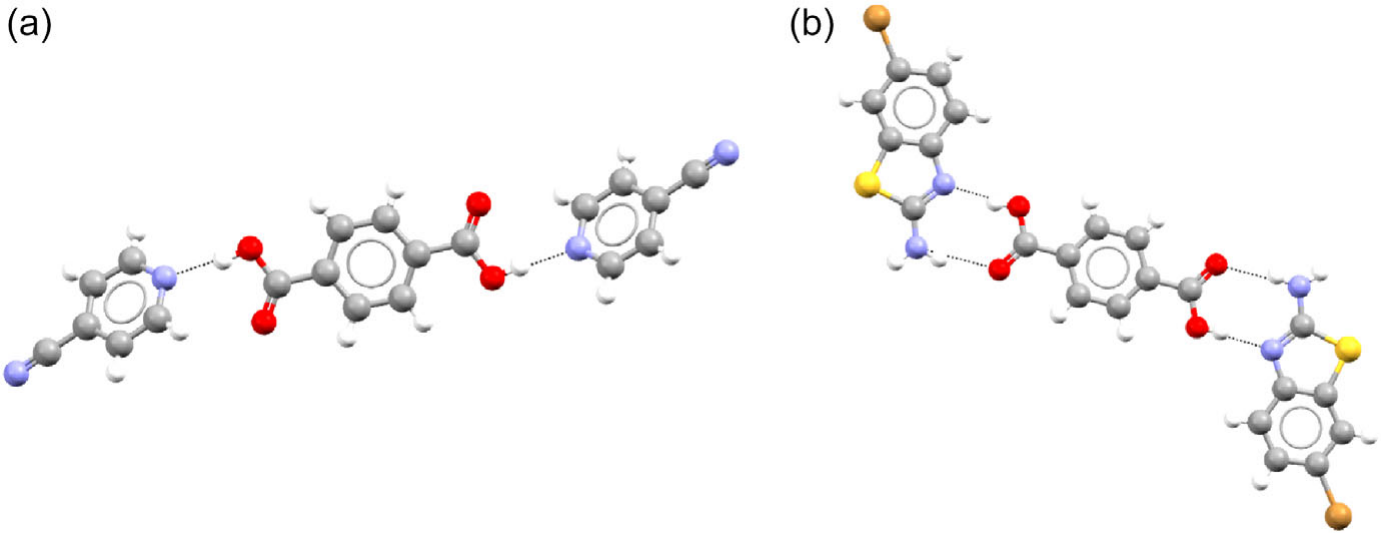
Terephthalic acid acting as a) a hydrogen bond donor (CSD refcode WEQHIN) and b) both hydrogen bond acceptor and donor (BASYUR01).

**Figure 8:**
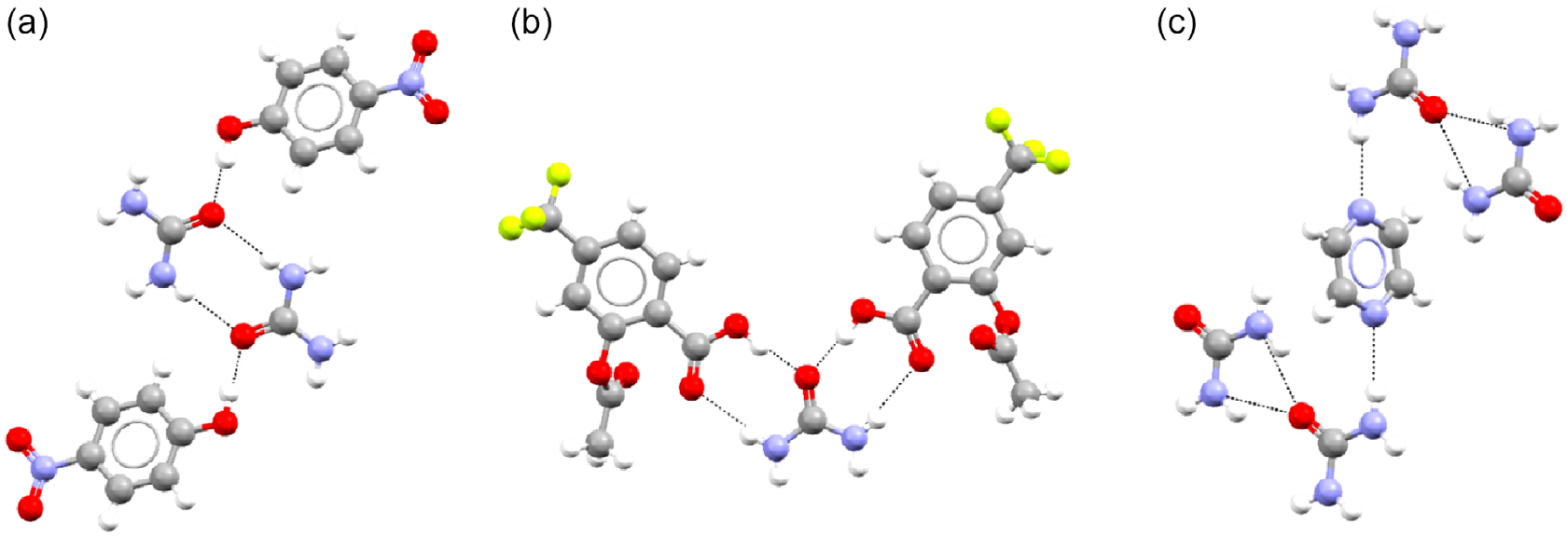
Urea acting as a) hydrogen bond acceptor (CSD refcode GAVHUH01), b) both hydrogen bond acceptor and donor (HABJUS), and c) hydrogen bond donor (QOFSIQ).

In order to understand the chemistry behind the clash between the bipartite structures of the two networks, we select examples of compounds with high and low bipartiteness scores in both networks. In previous work, we examined the most monopartite coformers in the cocrystal network.^[^ ^11^ ^]^ It was noted that a lot of the most monopartite coformers are molecules that can act as both a (hydrogen bond) donor and acceptor. This suggests that one bipartite group contains a lot of molecules that mainly act as (hydrogen bond) donors, and the other contains a lot of molecules that mainly act as (hydrogen bond) acceptors. As an example of this, we look at terephthalic acid (CSD refcode TEPHTH), a coformer with a bipartiteness of 1. In all of its cocrystals, it acts as a hydrogen bond donor. In a handful of cases, it acts as both donor and acceptor at the same time, but it never acts as an acceptor alone (see **Figure** **7**). On the other hand, we have examples of coformers that show clear monopartite behavior, like urea (UREAXX). Urea has several cocrystals where it acts as acceptor, donor, or both (see **Figure** **8**).

**Figure 9:**
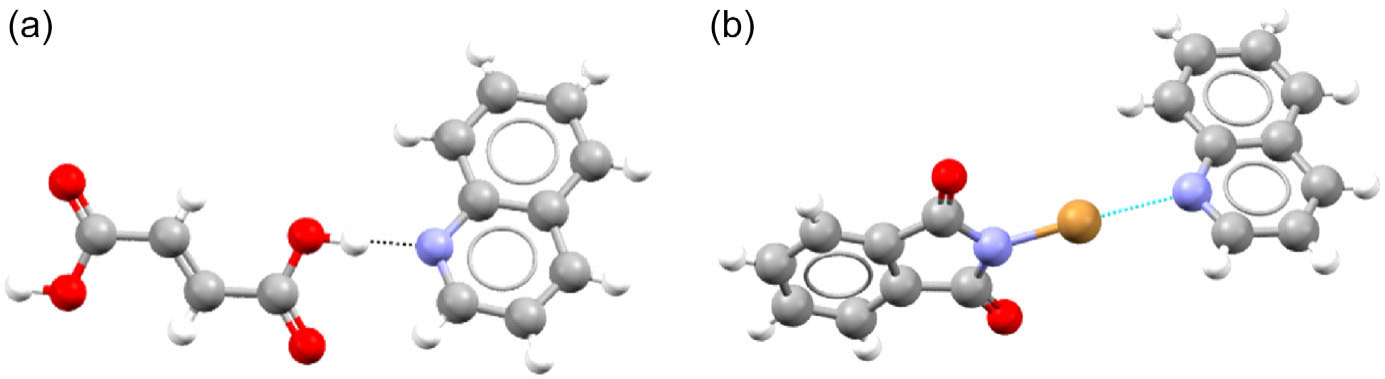
Quinoline forming a) a hydrogen bond (CSD refcode LASGOD) and b) a halogen bond (VELWER).

**Figure 10:**
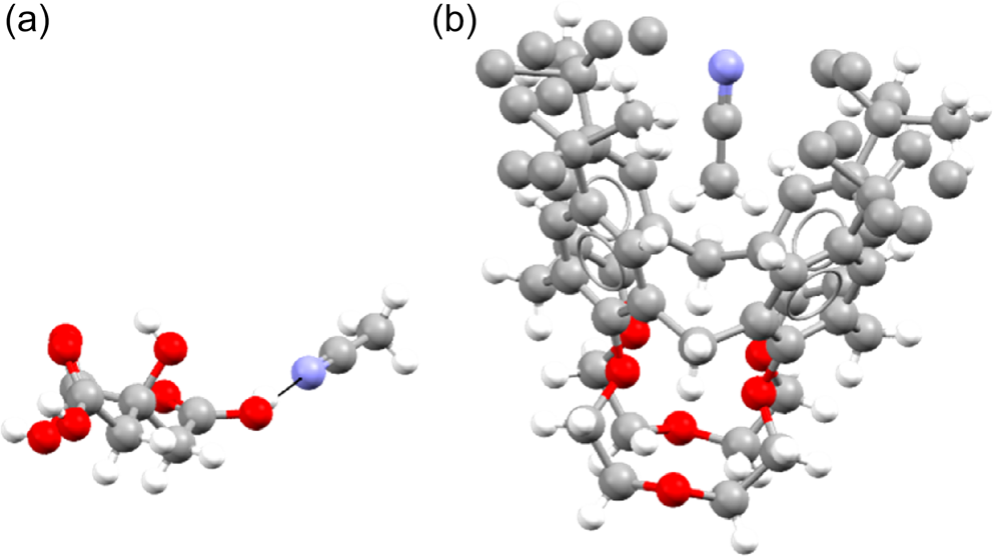
Acetonitrile acting as a) a hydrogen bond acceptor (CSD refcode QIZTEB) and b) a space filler (ISAWEE).

To see if the solvents exhibit the same behavior, we look at examples of bipartite and monopartite solvents. We will use quinoline (EDAVUA) as an example of a bipartite solvent and acetonitrile (QQQCIV) as an example of a monopartite solvent. We will examine the solvates that appear in the cocrystals_S network to find out why quinoline fits better into the bipartite structure of the cocrystal network than acetonitrile does (see Figure 6). Quinoline shows the expected bipartite behavior, as it acts as an electron donor in all of its solvates, either as a hydrogen bond acceptor (77%) or by forming a halogen bond (23%) (see **Figure** **9**). Acetonitrile, on the other hand, does something else. It does act as a hydrogen bond acceptor in 55% of its solvates with group *A* and 20% with group B, but it cannot act as a donor. Still, it is one of the most monopartite solvents (see Figure 6). The reason for this is that acetonitrile can take on another role: the role of “space filler.” Looking at its solvates, acetonitrile acts as a space filler in 80% of its solvates with group *B* coformers and 45% of its solvates with group *A* coformers (see **Figure** **10**). This is a kind of monopartite behavior we have not seen in the cocrystal network. It seems that while in the cocrystal network the two bipartite groups stemmed from chemical complementarity, in the solvate network, there is both chemical complementarity (e.g., hydrogen bonding or π‐stacking) and steric complementarity (space filling using only Van der Waals interactions). This competition between chemical and steric complementarity can result in different structural properties of the solvate network compared to the cocrystal network.

### 3.4 Biclique Analysis

Bipartiteness is an important structural feature of networks, but it is not sufficient to show meaningful structure in a network. One could imagine, for instance, a network consisting of pairs of nodes where each pair is linked to each other but not to any other node. Such a network would be perfectly bipartite but lack any interesting structure to speak of. Examining the degree distribution and k‐cores shows us more of the network's global structure. By examining the presence of so‐called maximal bicliques (see SI) using the algorithm designed by Zhang et al.^[^ ^17^ ^]^ we can learn more about the local structure of the networks.

**Figure 11:**
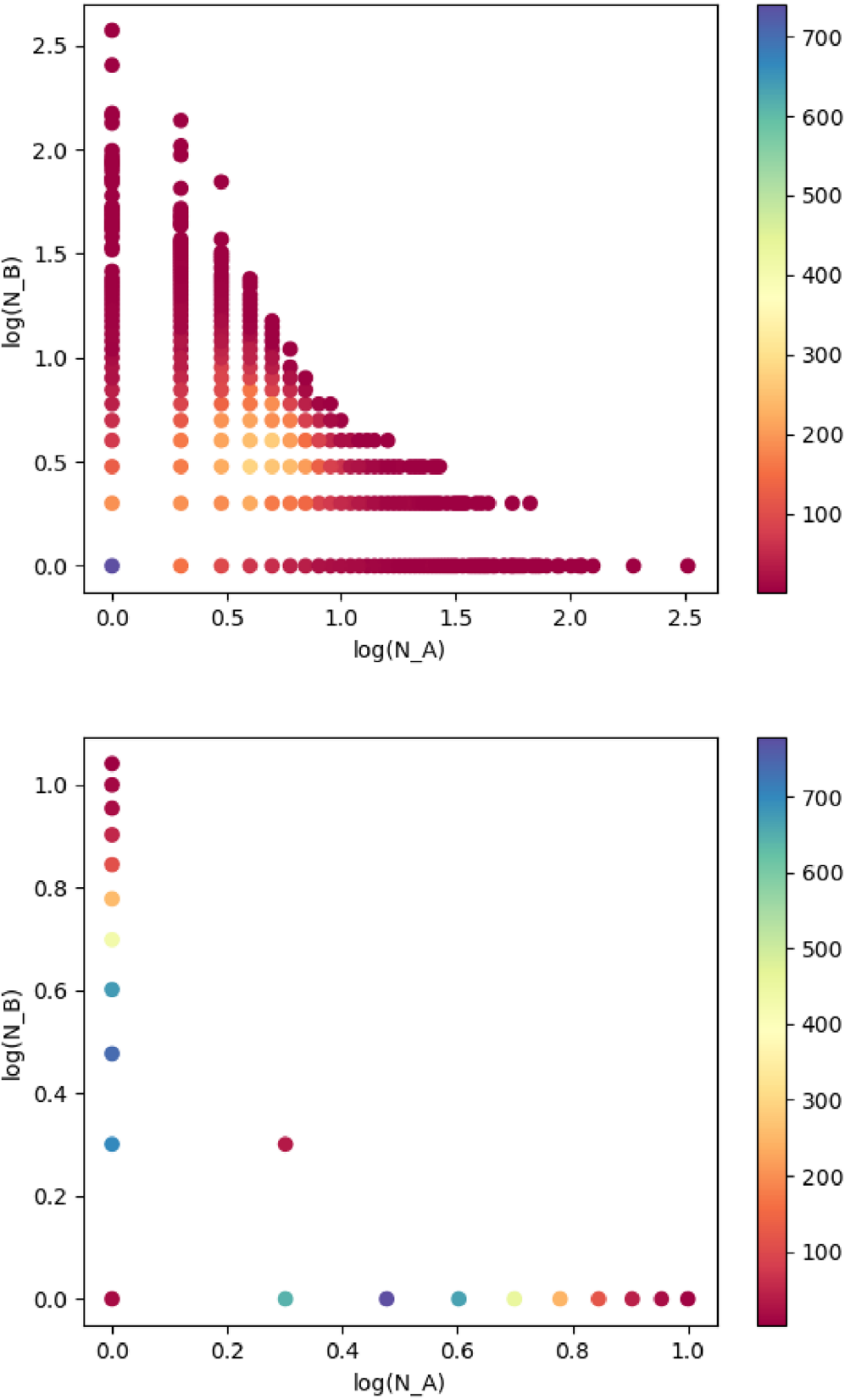
Color plots showing the number of bicliques per size for the cocrystal network (top) and a randomly generated network (bottom) of equal size.

First, looking at the entire networks: the cocrystal network contains 10 209 maximal bicliques with an average size of 5 × 5, and the solvate network contains 2491 maximal bicliques with an average size of 17 × 3. The solvate network has fewer bicliques, but they are bigger and more asymmetric. To get a better idea of the size distribution of the bicliques, we generate a scatterplot where the *x*‐ and *y*‐axes represent the dimensions of a biclique, and the color of a point represents how often bicliques with those dimensions appear. **Figure** **11** shows the biclique size distribution for the cocrystal network. For comparison, we also generate a network that has the same number of nodes and links as the cocrystal network but with randomly distributed links. As shown in Figure 11, the cocrystal network has a variety of clique sizes distributed evenly between the bipartite groups *A* and *B*. However, 1 × *n* and *n* × 1 bicliques are not of interest, as they only represent a single node with a degree of *n* and no further interesting structure around it. This means the only interesting feature in the random network is a small number of 2 × 2 bicliques. It is remarkable how many larger bicliques there are in the actual cocrystal network.

**Figure 12:**
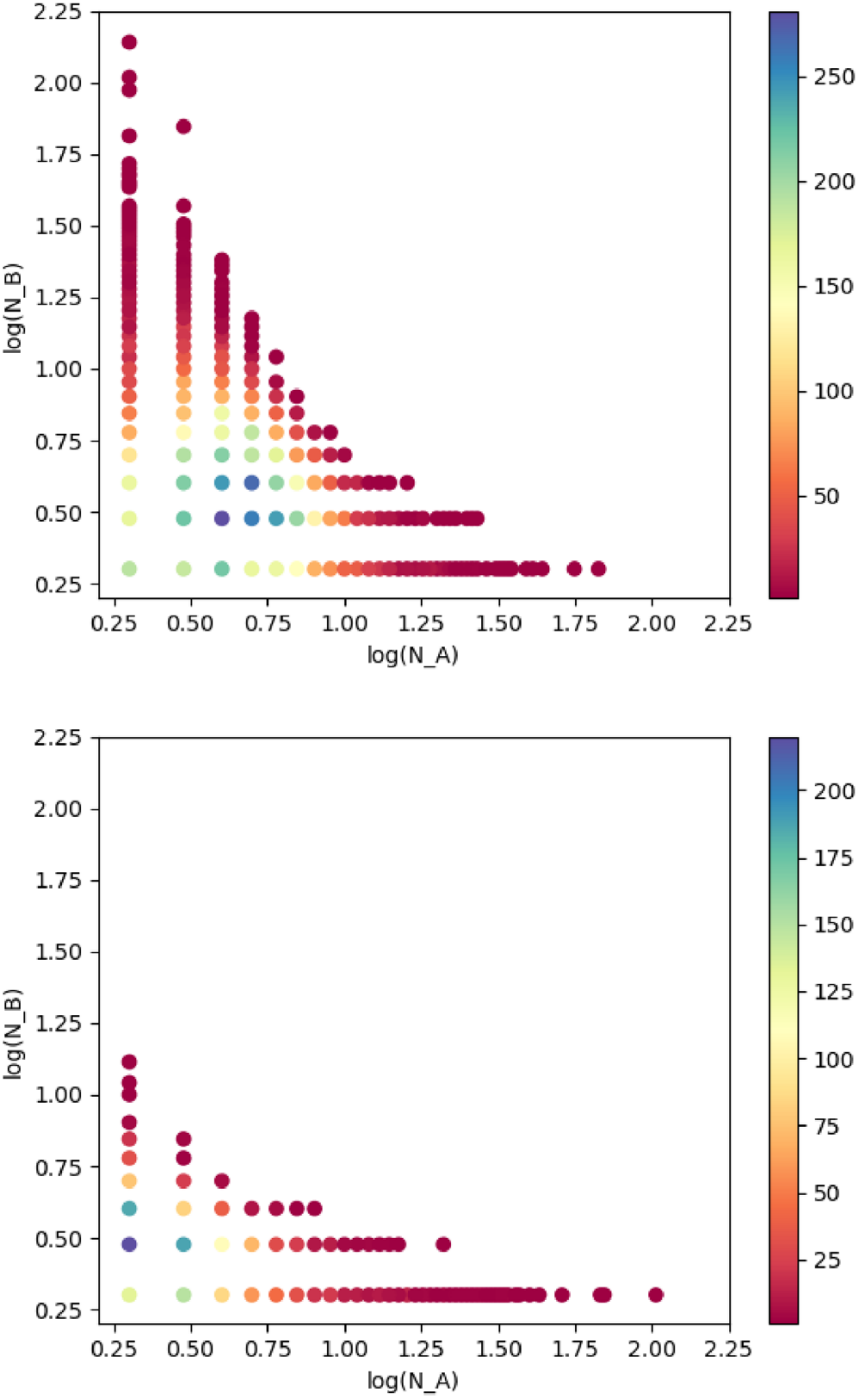
Color plots showing the number of bicliques per size for the cocrystal (top) and the solvate (bottom) networks, not including 1 × *n* and *n* × 1 bicliques.

When 1 × *n* and *n* × 1 bicliques are not counted, the cocrystal network contains 8120 maximal bicliques with an average size of 5 × 5, and the solvate network contains 1829 maximal bicliques with an average size of 5 × 3. The larger average size of the solvate bicliques we found earlier is thus largely due to individual nodes with very high degrees, in line with the degree distributions we discussed before. **Figure** **12** shows the biclique size distributions for the cocrystal and solvate networks, without the 1 × *n* and *n* × 1 bicliques. The solvate network has significantly fewer large bicliques, and the sizes are not evenly distributed between the two bipartite sets. The size imbalance can be explained by the fact that there are a lot more coformers than solvents in the network. The fact that the number of large bicliques in the solvate network is smaller despite the fact that the network itself is bigger suggests that the solvate network is not as well connected as the cocrystal network. Apparently, the solvate network is less saturated than the cocrystal network, and the fraction of undiscovered solvates is much larger than the fraction of undiscovered cocrystals. The presence of a large number of bicliques determines the success of link prediction algorithms. Given that the solvate network has fewer large bicliques, we expect it to show lower link prediction performance.

### 3.5 Link Prediction

**Figure 13:**
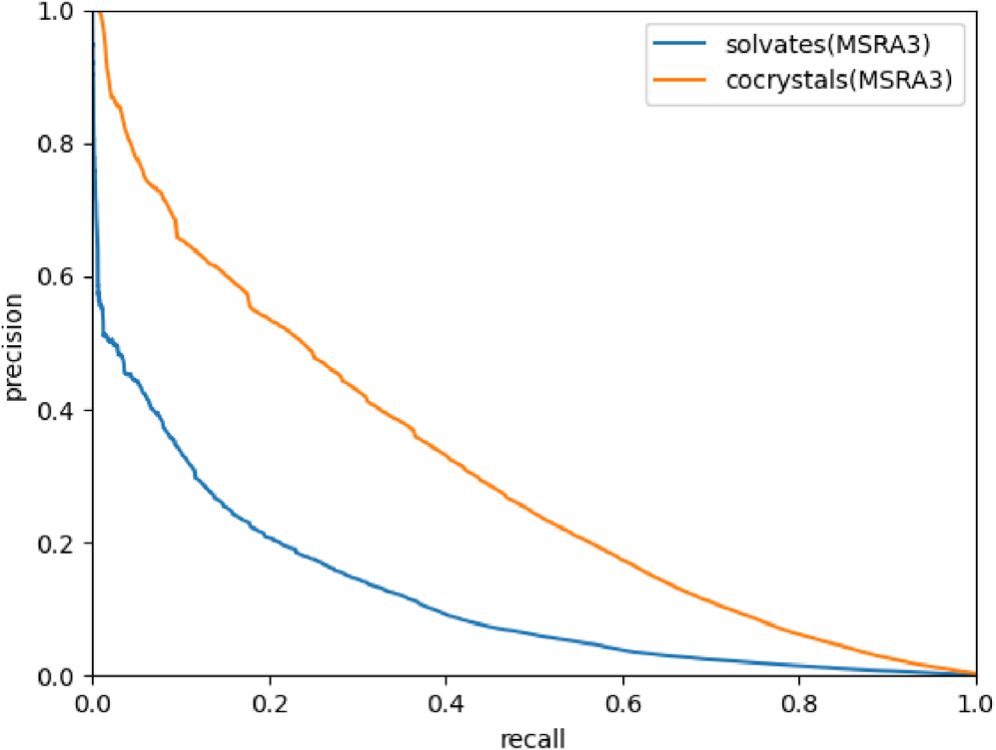
The precision–recall curves for link prediction with the solvate and cocrystal networks, both generated using the MSRA method.

In this section, we use the MSRA scoring function to plot a precision–recall (PR) curve for the solvate network and compare it to the PR curve for the cocrystal network. **Figure** **13** shows the PR curves for the solvate and cocrystal networks. As expected, the solvate network performs significantly worse. More importantly, the recall reaches zero well before the precision reaches one. This implies that the highest scores are given to nonexisting links.

**Figure 14:**
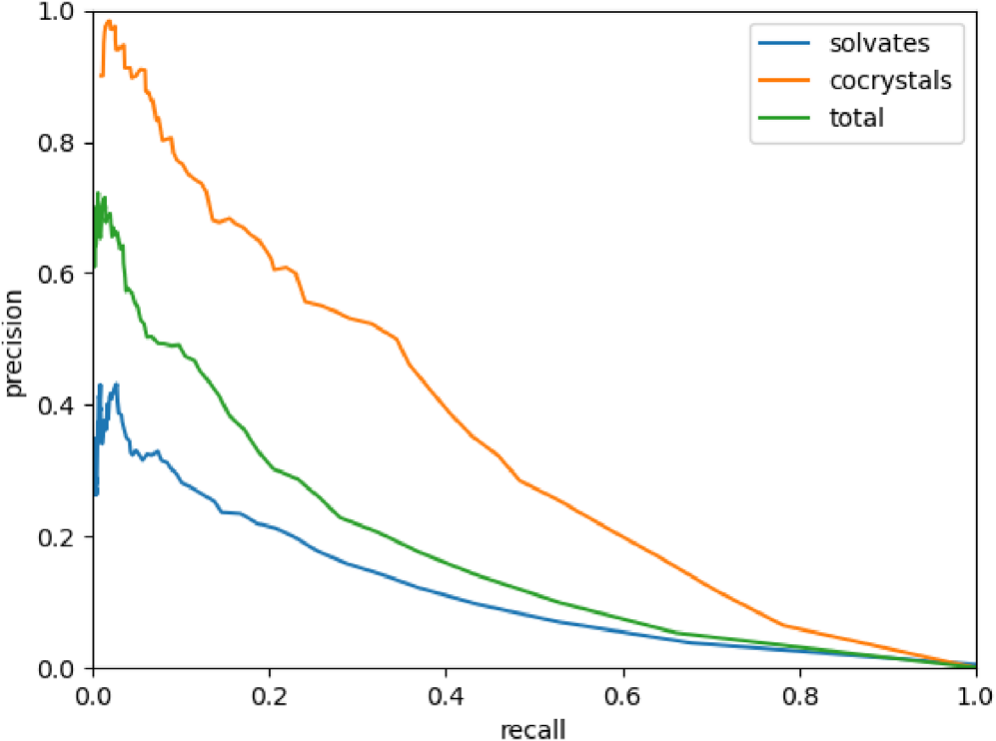
The PR curves for the Solvates_CC network. Since the Solvates_CC network can be used to predict both solvates and cocrystals, they are plotted separately.

It is possible that this solvate network performs poorly simply because it is undersaturated. We already saw that the link and biclique variety are less favorable in the solvate network. Some of the coformers in the solvate network can also form cocrystals together, but this information has been left out in the construction of the solvate network. We can improve the saturation of the solvate network by adding known cocrystal links between coformers in the solvate network to form the Solvates_CC network (see Methodology). Because there are cocrystal and solvate links in the Solvates_CC network, we can use it to predict both solvates and cocrystals. This means we can make three PR curves for the Solvates_CC network: one for the solvates, one for the cocrystals, and one for the total network. These PR curves are shown in **Figure** **14**.

Even with the cocrystal links added in, the solvate predictions are not improved. Apparently, the information in the cocrystal links does not improve prediction of solvates. The PR curve for cocrystals in the Solvates_CC network does not show the same problem. This, combined with the fact that the solvents showed an unusual degree distribution, suggests that some or all of the solvents behave differently from coformers.

One set of nodes that is potentially problematic is the set of “monopartite” nodes that do not fit into the bipartite structure of the network. We can use node bipartization (see SI) to remove these monopartite nodes.

To node bipartize the Solvates_CC network, 302 nodes needed to be removed (17% of nodes in the network, accounting for almost 50% of the links). We can plot the link prediction PR curves for the new node‐bipartized network and compare it to the original and link‐bipartized networks.

**Figure 15:**
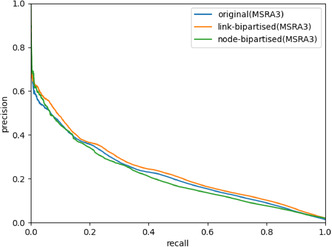
The PR curves for the original, link‐bipartized, and node‐bipartized Solvates_CC networks.

Despite the fact that the link‐bipartized version performs slightly better than the original network, it is plain to see in **Figure** **15** that the problem has not been solved yet. In fact, the node‐bipartized version performs slightly worse than the original. Node bipartization has not fixed the problems with the Solvates_CC network, perhaps because the high‐degree monopartite solvents influence the bipartization algorithm too much, causing it to remove the wrong nodes.

**Figure 16:**
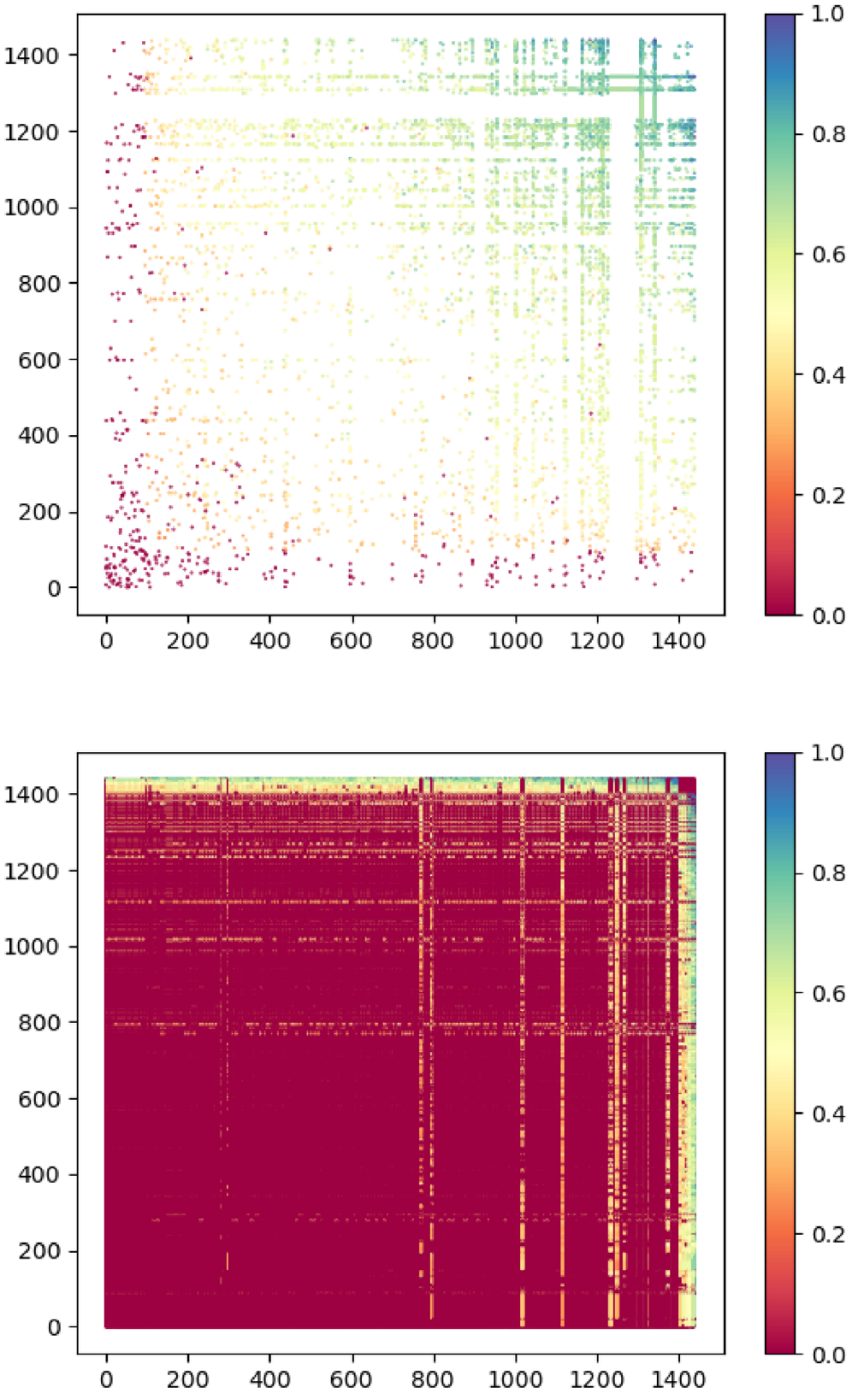
Scatterplot representations of the scores for linked pairs of nodes (top) and nonlinked pairs of nodes (bottom) in the solvate network. Node labels are assigned in order of average score.

So far, we used link prediction as a way of checking the predictive power of our networks. However, we can also use it to identify unusual nodes. To do this, we calculate the link prediction score for every pair of nodes in the regular solvate network. Pairs that share a link should score high on average, while pairs that do not should score low. These scores can be shown in a scatterplot where a link between a pair of nodes *n*, *m* can be represented by a point at positions (*n*, *m*) and (*m*, *n*). The relative score of each link is calculated using the three step version of the MSRA scoring function (MSRA3)^[^ ^18^ ^]^ and is represented by the color of the point. The nodes with degree 1 are not of interest. This is because MSRA3 requires a path of 3 links between two nodes to predict a link. Any link to a node with degree 1 is therefore guaranteed to get a score of 0. Because we are interested in nonzero scores, we choose to exclude nodes with a degree of 1 to increase the readability of the results. We plot the existing and nonexisting links separately in **Figure** **16**. In each of the plots in Figure 16, the nodes are plotted in order of increasing average score. This means the same node may have a different number in the top graph than it does in the bottom graph.

**Figure 17:**
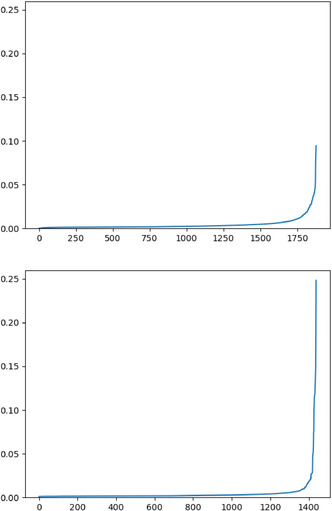
The average nonlink scores per node for the cocrystal (top) and solvate (bottom) networks.

As expected, most links have high scores compared to the nonexistent links. However, while the scores for linked pairs vary gradually, there are bands of nonexistent links at the top and right hand side of the graph where the scores increase dramatically. In other words, there is a small number of nodes for which the nonexistent link scores are unusually high. Plotting the average nonexistent link score per node for both the solvate and cocrystal networks (see **Figure** **17**) shows that the scores increase much more sharply and reaches higher values for the solvates. The shape of the PR curve of the solvate network in Figure 13 already suggested that nonexistent links receive higher scores than they should. Removing nodes in the solvate network with an average nonexistent link score over 0.1 (the highest average score found in the cocrystal network) could be a way to allow the solvate network to perform similarly to the cocrystal network.

**Figure 18:**
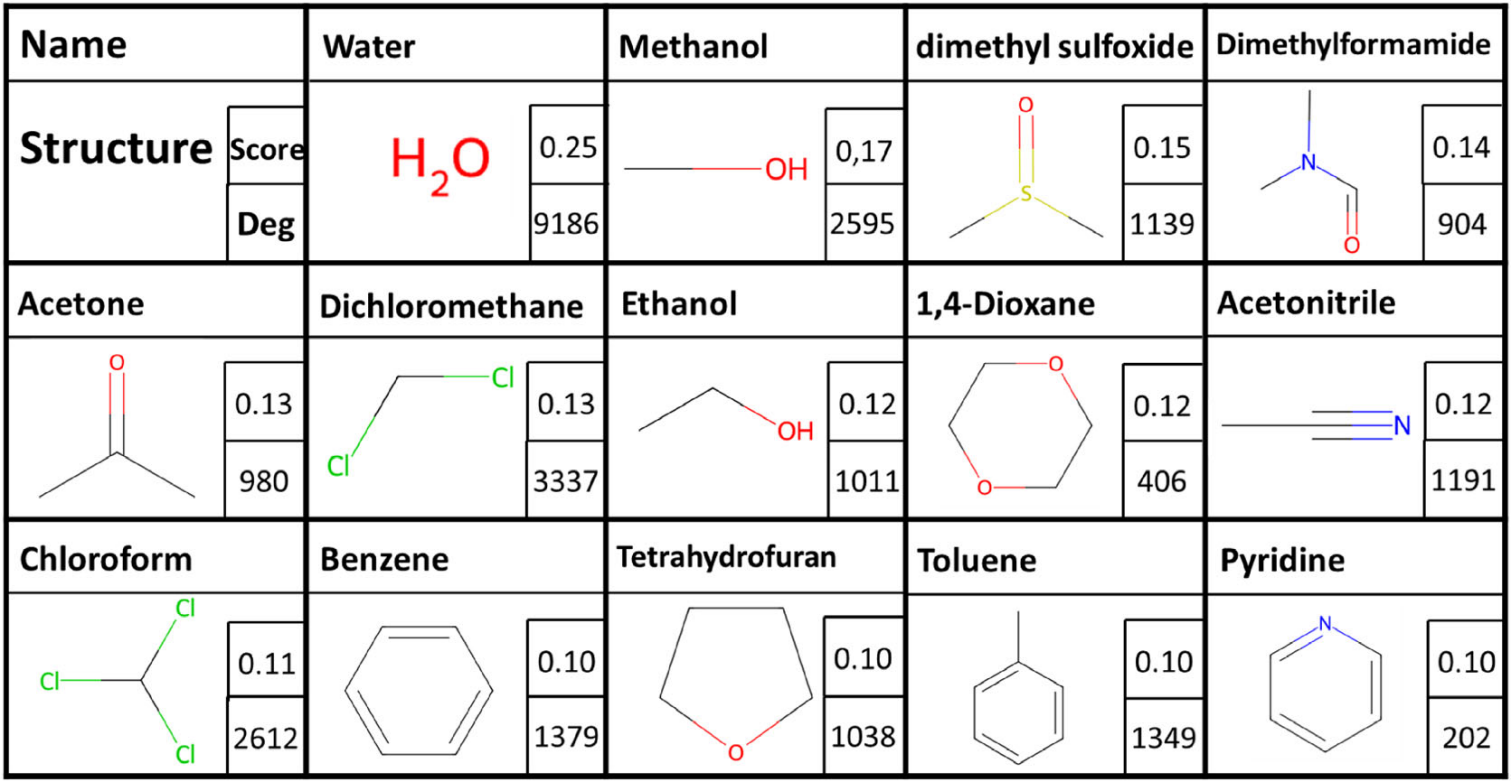
The 14 nodes with the highest average nonexistent link score.

There are 14 nodes in the solvate network with an average nonexistent link score of more than 0.1. These are shown in **Figure** **18**. All 14 nodes are solvents, which is in line with what we expected given the degree distribution in Figure 4. Remarkably, the 14 solvents are among the top 18 solvents most commonly found in organic solvates.^[^ ^12^, ^19^, ^20^ ^]^ Comparing Figure 18 to 6, the 14 nodes that we removed have a wide range of bipartiteness scores. However, the solvents with higher link prediction scores for nonexistent links have lower bipartiteness scores, which shows there is a connection between monopartiteness and unusual link prediction behavior.

**Figure 19:**
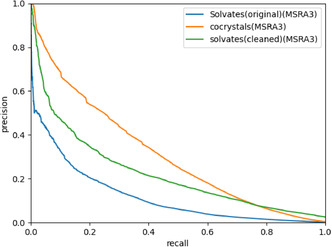
The PR curves of the cocrystal network, the original solvate network, and the cleaned solvate network.

If these 14 nodes do indeed account for the unusual behavior, we expect that the link prediction PR curve should improve significantly after they are removed. We removed these 14 nodes from the solvate network, along with any nodes that became disconnected from the rest of the network after the first 14 were removed. We plot the PR curve of this “cleaned” solvate network along with those for the cocrystal and original solvate networks (see **Figure** **19**). This shows that while the cleaned solvate network still does not perform as well as the cocrystal network, the PR curve has gone up significantly and no longer reaches a recall of zero before reaching a precision of one. The 14 solvents we removed reduced the link prediction performance instead of adding valuable information to the network. The fact that the cleaned solvate network still does not perform as well as the cocrystal network is related to the smaller size (5354 nodes and 6040 links) and the poorer biclique structure of the solvate network, both of which show that the solvates are undersampled in the CSD compared to the cocrystals. The 14 removed solvents accounted for over three quarters of all links in the solvate network, most of which appear to be antennae. Usually, removing information from a network decreases the predictive power. The fact that we see an increase instead means that the part of the network we removed was not in accordance with the rest of the network. Our reason for removing exactly 14 solvents, which was based on making it more similar to the cocrystal network, was somewhat ad hoc. To ensure that it is the correct number, we varied the number of removed nodes from 10 to 20 and found that 14 is indeed the optimum (see SI).

Van der Sluis et al.^[^ ^12^ ^]^ examined the roles of the 15 most common solvents in the formation of solvates. Because the 14 solvents we removed are in that list, we can examine their roles to look for a pattern. All but one of the 14 solvents can act as a space filler or can take on multiple different roles. These solvents can break the rules of chemical complementarity either by using steric complementarity instead or by taking multiple roles. This leads to a lack of selectivity in coformers for these solvents. There are several examples of coformers like cholic acid that can form solvates with almost all of the 14 solvents, despite the fact that the solvents have different roles. According to Van der Sluis et al.^[^ ^12^ ^]^ the only exception is dimethylformamide (DMF). However, when we examined some solvates of DMF in the CSD, we also found examples where DMF appears to act solely as a space filler (e.g., CECFOI^[^ ^21^ ^]^), indicating that it is more versatile than previously thought.

Their double roles and space filling capabilities allow these 14 solvents to break chemical complementarity, leading to decreased prediction accuracy. This lack of selectivity also explains their frequent occurrence in solvates.

**Figure 20:**
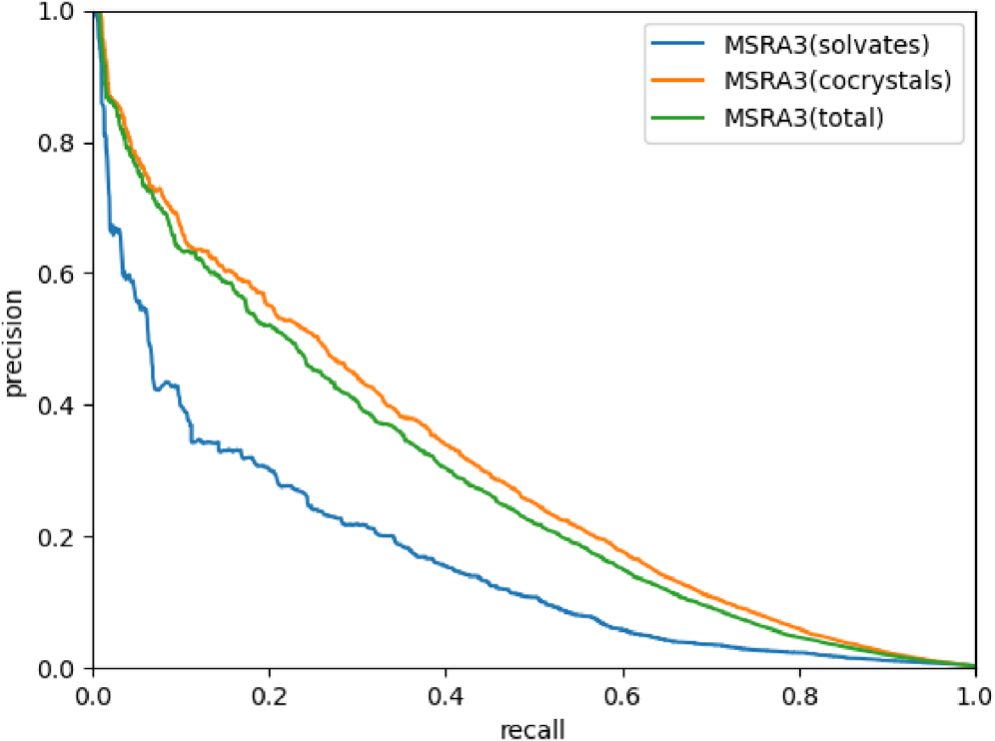
PR curves for solvate links (blue), cocrystal links (orange), and all links (green) in the mixed network.

Now that we can perform link prediction on the solvate network reasonably well, it is worth examining if we can create one mixed network that combines the cocrystal network and the cleaned solvate network (so without the solvents that we removed previously). We can use this mixed network to see if cocrystal prediction can be enhanced by including solvates and vice versa. This will also tell us if solvates and cocrystals can be treated equally or if they need to be treated separately. **Figure** **20** shows the PR curves for the mixed network. Because this network contains both solvate links and cocrystal links, we again plot separate PR curves for solvates and cocrystals in this mixed network, as well as the PR curve for the entire mixed network. Comparing Figure 20 to 19, we can see that the predictions for both solvates and cocrystals are roughly the same in the mixed network as they were in their respective networks. This shows that information from one network does not directly contribute to predictions in the other, even after we removed the 14 solvents.

## 4 Conclusion

We found that the solvate network is several times larger than the cocrystal network, but also significantly less dense. The networks share a relatively small overlap of ≈900 nodes. The degree distributions showed a different network structure for solvents than for coformers, and by examining the k‐core sizes, we found that the solvate network is less well connected. While the solvate network does have its own inherent bipartite structure, it clashes with that of the cocrystal network. This can be explained by a competition between chemical and steric complementarities during solvate formation. Biclique analysis showed in more detail that the solvate network lacks the well‐connected structure found in the cocrystal network. Our link prediction algorithm performs poorly when applied to the solvate network, compared to the cocrystal network. By looking for nodes that showed unusually high scores for nonexistent links, we were able to identify 14 solvents that are responsible for reducing the link prediction performance. These 14 solvents subvert the chemical complementarity required for network‐based link prediction. As a result, they are much less selective than the rest of the solvents, causing them to skew the predictions. Combining the cocrystal and solvate networks showed that information from one network does not contribute to predictions in the other.

We set out to answer the question: are cocrystals and solvates the same? We can speak only from the perspective of networks and the data currently available in the CSD. From this perspective, the answer is no. The clashing bipartite structures and failure of the mixed network at increasing link prediction accuracy both imply that solvates and cocrystals must be treated separately. Furthermore, the k‐core and biclique analyses show that the solvate network is still underdeveloped when compared to the cocrystal network. There is plenty of room for discovery in the solvate landscape, and large‐scale solvate screening is required to develop a more complete solvate network comparable to the cocrystal network.

## Conflict of Interest

The authors declare no conflict of interest.

## Supporting information

Supplementary Material

## Data Availability

The data that support the findings of this study are available from [Cambridge Crystallographic Data Centre]. Restrictions apply to the availability of these data, which were used under license for this study. Data are available from the authors with the permission of [Cambridge Crystallographic Data Centre].
