# Supplementary material for: Cocrystals and Solvates are Not the Same: A Network Perspective
Source: Chemphyschem. 2025 Oct 1;26(23):e202500338. doi: 10.1002/cphc.202500338 (PMC12677714; doi:10.1002/cphc.202500338)
Supplement: Supplementary file 1 — Supplementary Material [file CPHC-26-e202500338-s001.pdf]

Supporting Information: "Cocrystals and Solvates are Not the Same: A Network Perspective."

Tom E. de Vries[1], Elias Vlieg[1], Hugo Meekes[1], and René de Gelder[1,*]

[1]*Radboud University, Institute for Molecules and Materials, Solid State Chemistry, Heyendaalseweg 135, 6525 AJ Nijmegen, The Netherlands, *email: r.degelder@science.ru.nl*

Contents

S1 Network construction **1**

S2 Degree distribution and k-core analysis **3**

S3 K-core sizes compared to random networks **4**

S4 Bipartisation **4**
S4.1 Link bipartisation 5
S4.2 Node bipartisation 5

S5 Biclique analysis **5**

S6 Link prediction **6**

S7 PR-curves for varying numbers of removed solvents **7**

S8 List of solvents in the CSD **8**

S1 Network construction

The CSD python API[1] (Application Programming Interface, not to be confused with Active Pharmaceutical Ingredient) is used to extract all CSD entries that are organic, have no errors, are not polymeric or ionic, contain exactly two different residue types, and have their three-dimensional coordinates determined. The list of entries is then separated into solvates and cocrystals using a custom classifier algorithm written by Devogelaer et al.[2] A unique canonical SMILES code is generated for each MCC. In canonical SMILES codes, different molecular components are separated by a "." character, making it easy to split the MCC into its constituent components. Two networks are created, one for solvates and one for cocrystals. In each network, a node is created for every compound. The nodes are marked as either coformer (for solid components) or solvent (for liquids). The distinction is made using a list of canonical SMILES codes of known solvents that appear in the

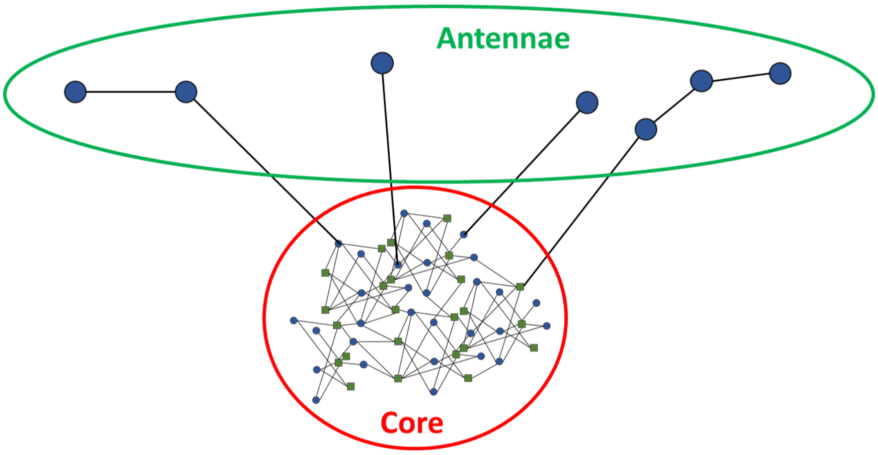

Figure S1: The network consists of a core of well-connected nodes, and antennae, which only connect to the rest of the network through a single line of links.

CSD (see section S8). For every solvate or cocrystal, a link is created between the nodes representing its two constituent components. The networks are stored in an $N \times N$ adjacency matrix A, where N is the number of nodes and $A_{i,j} = 1$ if there is a link between nodes i and j, and $A_{i,j} = 0$ otherwise.

All the networks we have created consist of a relatively dense 'core' and a large number of what we call 'antennae': nodes that are only connected to the network via a single line of links (see figure S1). These antennae do not contribute to link prediction or bipartisation. They do, however, greatly increase the runtime of any network analysis algorithm. While these antennae do contribute to the structure of the networks, they can make it difficult to examine certain properties, because there are so many of them. For these reasons we will occasionally 'prune' our networks. This means we check the number of links of all the nodes in the network, remove any node that has exactly one link, then recalculate the number of links for the remaining nodes. This is repeated until there are no nodes with one link and only the so-called 2-core of the network remains (see section S2).

S2 Degree distribution and k-core analysis

To analyse the global structure of a network, the degree distribution of a network can be calculated. The degree of a node is defined as the number of links connected to that node. Using the adjacency matrix, it is easy to calculate the degree of node n (deg(n)):

$$deg(n) = \sum_{m=1}^{N} A_{n,m} \tag{1}$$

We can plot the degree distribution of the network by plotting the number of nodes with degree d (N_d) against d. Devogelaer et al.[2] previously reported that the degree distribution of the cocrystal network follows a power law, which indicates that it is a scale-free network. To check whether the solvate network follows a similar power law, and to more easily visualise and compare the distributions, they are plotted on a log-log scale.

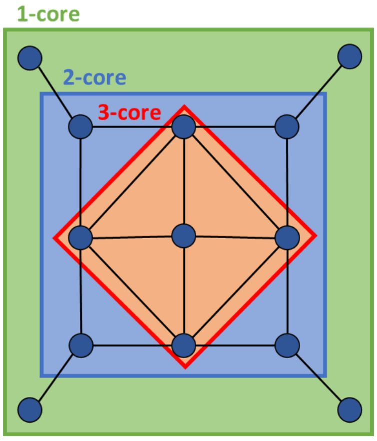

Figure S2: An example network with the 1-, 2-, and 3-cores marked.

Another tool for examining of global network structure are so-called k-cores. A k-core is a connected subset of a network such that every node in the k-core has at least k links to other nodes in the k-core. It is possible for a network to have multiple disconnected k-cores for any given k. For the sake of simplicity, we will make use of 'maximal' k-cores: the *largest possible* subset of a network such that every node in the maximal k-core has at least k links to other nodes in the maximal k-core (unlike a regular k-core, this does not need to be a connected subnetwork). By this definition, the maximal k-core is simply the union of all k-cores. Our networks have no nodes with a degree of 0, so the maximal 1-core is simply the entire network. Calculating a maximal k-core (for $k > 1$) is done by calculating the degree of every node in the network, and then removing those with degree 1 or less. This will lower the degree of other nodes, so the degrees are recalculated. Again, nodes with degree 1 or less are removed. This process is repeated until no nodes with degree 1 remain. The resulting network is the maximal 2-core of the original (this is equivalent to the pruned network described in section S1). To find the maximal 3-core, the previous process is repeated by now removing nodes with degree 2 or less. This continues until the k-core is found, or the network is empty. An example network is shown in figure S2.

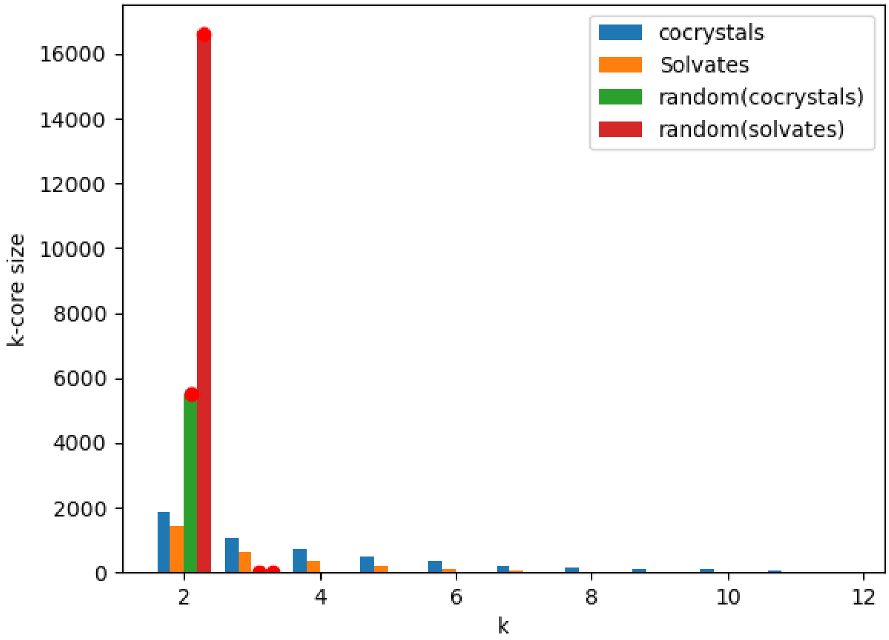

Figure S3: The k-core sizes of the cocrystal and solvate networks compared to the average k-core sizes of 100 random networks with the same number of nodes and links as the cocrystal and solvate networks.

S3 K-core sizes compared to random networks

Figure S3 shows the k-core sizes for the cocrystal and solvate networks, along with the average k-core sizes of 100 randomly generated networks with the same size as the cocrystal and solvate networks. The standard deviation of the 2-core size is approximately 32 for the cocrystals, and 81 for the solvates. Not a single one of the random networks had a 3-core size above 0. This shows that the cocrystal and solvate networks have a larger number of 'antennae' than expected in random networks of their size, because their 2-core is significantly smaller. However, they are also significantly more well connected than random networks, because they actually have a non-zero 3-core and beyond.

S4 Bipartisation

In mathematical terms, a network with nodes N and links L is bipartite if and only if it is possible to define two sets of nodes $\{N_A, N_B\}$ with $N_A \cup N_B = N$ and $N_A \cap N_B = \emptyset$ such that for all links $(l_1, l_2) \in L$, if $l_1 \in N_A$ then $l_2 \in N_B$ and vice versa.
We will examine whether a network has a bipartite structure using a method called bipartisation. Bipartisation is the process of taking a network that is not bipartite, and turning it into a bipartite network while removing as little as possible. The two most common ways of bipartising a network are removing links (link bipartisation) or removing nodes (node bipartisation).

S4.1 Link bipartisation

In previous work, we described a near-optimal link-bipartisation algorithm.[3] This algorithm can link-bipartise large networks quickly. It does so by counting the number of 3-cycles and 4-cycles that a link is part of. An n-cycle is a cyclic path $A - B_1 - ... - B_{n-1} - A$ consisting of n links. In a bipartite network, 3-cycles cannot exist, and 4-cycles are expected to be common. Thus, to bipartise a network, the algorithm starts by removing all 3-cycles. This is done by giving the three links in each 3-cycle a 'bipartiteness' score. The bipartisation algorithm calculates this score for each link by subtracting the number of 3-cycles it is a part of from the number of 4-cycles, and removes the link with the lowest score.

If the network is not yet bipartite after all 3-cycles have been removed, the algorithm calculates the bipartiteness score of every link in the network and removes the lowest scoring link. This is repeated until the network is fully bipartite. To avoid removing too many links, the algorithm attempts to replace each removed link in order of the bipartiteness score they had at the start of the process. If replacing the link does not break the bipartisation, it is kept. Otherwise, it is discarded.

Unlike exact bipartisation algorithms, it is possible that this algorithm removes more links than necessary, but exact algorithms are too slow to use on a network of the size used here. After the bipartisation is done, we can use the number of links that have been removed as a measure of how bipartite the original network was.

S4.2 Node bipartisation

After the link-bipartisation algorithm has finished, we know which links do not belong to the bipartite structure of the network (so-called monopartite links). We can use the list of monopartite links, obtained from the link-bipartisation algorithm, to perform node-bipartisation. The node-bipartisation algorithm looks at the list of monopartite links and calculates a new bipartiteness score by subtracting for every node the number of monopartite links from the number of bipartite links and dividing by the degree of the node. In this way, the most monopartite node can be identified. This node and its links are temporarily removed from the network. After the list of monopartite links is updated, the next most monopartite node is determined and removed. This process is repeated until the network is fully bipartite. To avoid removing too many nodes, the algorithm next attempts to replace each removed node, running from the least to the most monopartite node in the original list. If the network is still bipartite after a node is replaced, the node is kept. If the network is not bipartite, the node is removed permanently. Node-bipartisation can be a useful tool for identifying nodes that behave differently from the other nodes in a network.

S5 Biclique analysis

A clique is defined as a group of nodes C where every node in C is linked to every other node in C. Because the networks discussed in this work are (nearly) bipartite, we will use a variant of cliques called bicliques. A biclique consists of two sets of nodes C_A and C_B such that every node in C_A is linked to every node in C_B and vice versa. The sets C_A and C_B are always subsets of the two bipartite sets in the network. We refer to the size of a biclique as $N_A \times N_B$ with N_A the number of nodes in set C_A and N_B the number of nodes in set C_B. In principle, any pair of linked nodes forms a 1×1 biclique, even if the pair is also part of a larger biclique. We are most interested in so-called maximal bicliques. A maximal biclique is a biclique that is not a subset of a larger biclique. Figure S4 shows an example network with a 2×3 maximal biclique (green), a 3×1 maximal biclique (blue), and a 2×2 non-maximal biclique (red).

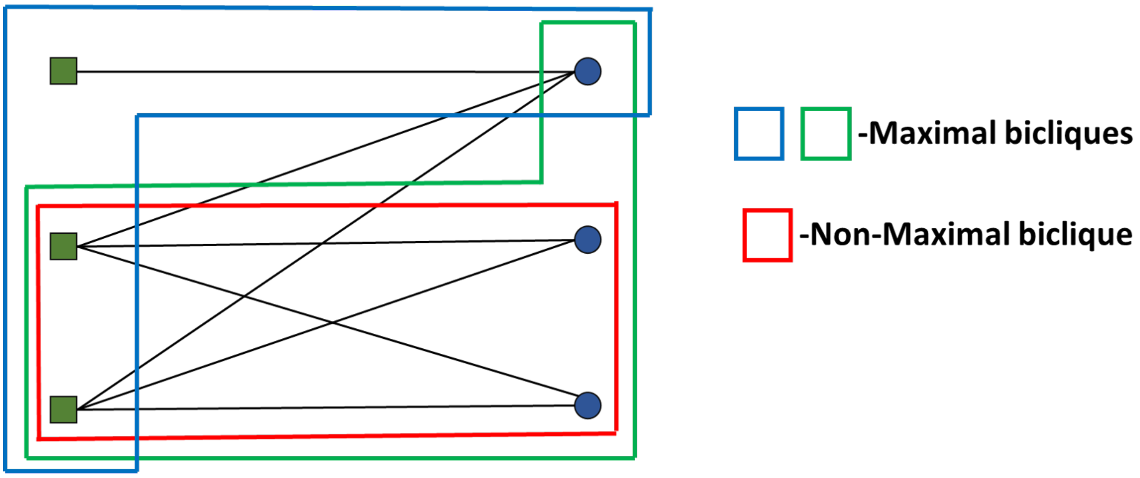

Figure S4: Examples of maximal and non-maximal bicliques. The red biclique is not maximal because it is a subset of the green biclique.

The presence of bicliques is also important for link-prediction algorithms (see section S6). They can be used directly to make predictions,[4] but many algorithms use them indirectly, or require their presence to make predictions. We use the algorithm designed by Zhang et al.[5] to find all maximal bicliques in the solvate and cocrystal networks. We can subsequently examine the structure of the networks by looking at the number of maximal bicliques, as well as their sizes.

S6 Link prediction

There are many different scoring functions, and the scoring function called Multi-Step Resource Allocation (MSRA) was previously found to be the most accurate one for the cocrystal network.[6] The MSRA score is based on counting paths of various lengths between two target nodes. It was found that counting three-step paths (MSRA3) resulted in the most accurate predictions. To calculate the MSRA3 score for a pair of nodes (i, j), the algorithm first identifies the neighbours of i and j. It then checks if any of the neighbours of i link to the neighbours of j. If so, there is a path with a length of three steps from i to j. Each of these paths $i - n_i - n_j - j$ is then given a score of $\frac{1}{d(n_i)d(n_j)}$ where $d(n)$ is the degree of node n. The scores for all three-step paths are added together to obtain the final score for the link (i, j).
MSRA does not use bicliques directly, but their presence is important. For MSRA to give a high score to a pair of target nodes, a lot of paths between them need to exist. If there are a lot of bicliques between the targets, then there will also be a lot of paths between them, thus increasing their MSRA score.

To test the accuracy of a link-prediction model on a particular network, Precision-Recall (PR-)curves are used. To calculate the PR-curve, we first randomly select 10% of the links in the network for our test set, these links are removed from the network. We also randomly select 10% of the non-existent links in the network. This process is repeated 10 times such that every link and every non-existent link appears in a test set once. We then use the model to calculate the score of all the links and non-existent links in the test set. A variable threshold score t is then varied from zero to the highest score. For each value of t, every (non-existent) link with a score $s \geq t$ is given a positive prediction,

every (non-existent) link with a score $s < t$ is given a negative prediction. We then count the number of True Positive (TP), True Negative (TN), False Positive (FP), and False Negative (FN) predictions for each value of t (see figure S5).

Truth ↓ \ Prediction →	Positive	Negative
Positive	True Positive (TP)	False Negative (FN)
Negative	False Positive (FP)	True Negative (TN)

Figure S5: The table of confusion. This table describes the results of a predictive model when compared to reality.

Using these numbers we can calculate the precision and recall of the model:

$$Precision = \frac{TP}{TP + FP} \qquad Recall = \frac{TP}{TP + FN}. \tag{2}$$

By plotting the value of the precision and recall for each value of t and average over the 10 test sets, we obtain the PR-curve.

To make sure that MSRA3 is also the best scoring function for the solvate network, we compare the PR curve for MSRA3 to a number of other scoring functions in figure S6. MSRA clearly outperforms the other methods, which shows that it is the best scoring function for the solvate network as well. We do not take link-prediction scores of 0 into account when calculating the PR curves because the validation method we use can cause certain links to become impossible to predict. This can happen because we remove the links chosen for testing. Coformers can be cut off from the network completely and the link-prediction algorithm is then unable to predict links to these coformers. Including scores that were incorrectly marked as 0 would skew the results by artificially increasing FN. The scores that are marked correctly as 0 only affect TN, which does not appear in the expressions for either the precision or recall, and removing them will have no effect on the PR-curves. This also means that using a pruned network will not affect the PR-curves.

S7 PR-curves for varying numbers of removed solvents

Figure S7 shows the PR-curves for the solvate network with a number of removed solvents ranging from 10 to 20. The network with 14 solvents removed shows the best PR-curve.

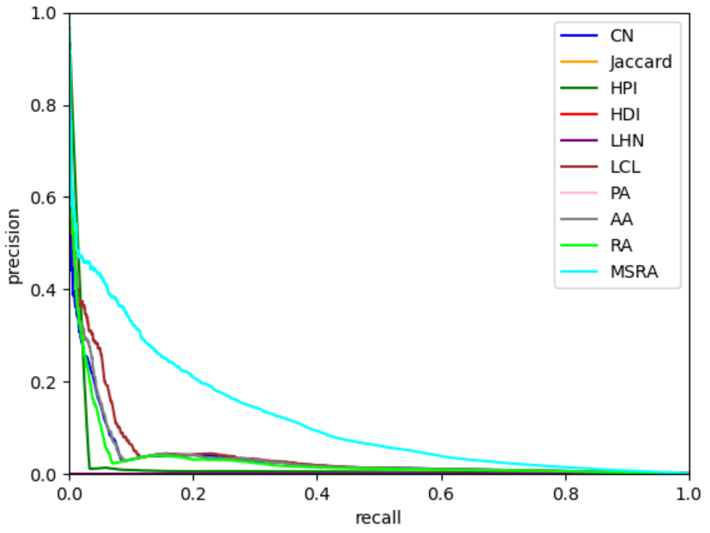

Figure S6: PR-curves for various scoring functions on the solvate network

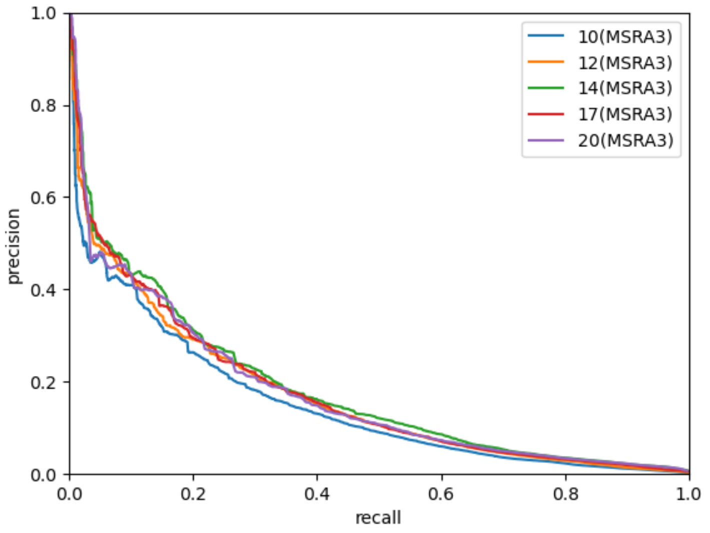

Figure S7: The PR curves for the solvate network with 10, 12, 14, 17, and 20 solvents removed.

S8 List of solvents in the CSD

This is a list of canonical SMILES codes for known solvents in the Cambridge Structural Database. This list was used to classify multi-component crystals as either cocrystals or solvates.

Clc1ccccc1Cl
CCN(CC)CC
Fc1c(F)c(F)c(c(c1F)F)F
O=C1C([2H])([2H])C([2H])([2H])C(C(C1([2H])[2H])([2H])[2H])([2H])[2H]
[O-][N+](=O)CCC
CC(=O)C(=O)C
Clc1cccc(c1)Cl
OC1CCCCC1
BrC(Br)Br
CCCCCCCO
CCS
C1CCCCCCC1
CCO
Nc1ccccc1
Brc1cccc(c1)Br
CC(O)(C)C
C1CCCCCC1
ClCC(CCl)Cl
OCc1ccccc1
Cc1ccc(cc1)C
CC1=CCC(CC1)C(=C)C
O=C1CCC1
CCOCCOC(=O)C
O1CCOCC1
CCSCC
CN1CCN(CC1)C
CCCCCCCCCCc1ccccc1
O([2H])[2H]
C([2H])(N(=O)=O)([2H])[2H]
CCCCCCCCCCCC
CC[C@H](O)C
CCCCCO
Fc1cc(F)cc(c1)F
CCCCO
N#Cc1ccccc1
CCCCCC
OCC(F)(F)F
CCCCC
CCOC(OCC)OCC
Cc1ccncc1
C([2H])(Br)(Br)[2H]
CCC(=O)O
c1ccco1
O([2H])C(=O)C([2H])([2H])[2H]
OC(=O)C(F)(F)F
CC(Cl)Cl
NC1CCCCCCC1
O=C1CCCC1

COC(=O)C=C
CCCCBr
OC(=O)C=C
CCOC=O
OCC(CO)O
NC=O
Cl/C=C/Cl
Fc1ccccc1F
O=C1CCCCC1
CCNCC
OO
CCCC(=O)O
C=CC#N
C=CCC=C
N#CCCCCC#N
c1([2H])c([2H])c([2H])c(c(c1C([2H])([2H])[2H])[2H])[2H]
CCCCCC(C)C
CCCC(=O)C
Cc1ccnc(c1)C
C1COOC1
CCCCCCN
Fc1c(F)c(F)c(c(c1F)C(F)(F)F)F
C[Si](O[Si](C)(C)C)(C)C
Cc1cnccn1
C1([2H])([2H])OC(C(C1([2H])[2H])([2H])[2H])([2H])[2H]
ClCCl
c1([2H])c([2H])c([2H])c(c(c1[2H])[2H])[2H]
CC(Br)(C)C
CC(=O)OC(C)(C)C
Brc1ccccc1Br
OCCCO
CCC(=O)OC
OC(C(F)(F)F)C(F)(F)F
CC(C(C)(C)C)C
O=S1(=O)CCCC1
CC1CCCO1
ICI
[O-][N+](=O)C
CN1CCN(C1=O)C
C1CCCC1
CCCC#N
ClC(C(Cl)Cl)Cl
CP(CCP(C)C)C
[O-][N+](=O)O
C1([2H])([2H])C([2H])([2H])C([2H])([2H])C(C(C1([2H])[2H])([2H])[2H])([2H]) [2H]
CCC(O)(C)C
O=CN(C)C
Cl/C=C\Cl
Cc1cccnc1

CC(CC(=O)C)C
CCCCCCCO
OCCCCCO
FCC1(CF)C(CF)(CF)C(CF)(CF)C(C(C1(CF)CF)(CF)CF)(CF)CF
ClS(=O)Cl
ClC(=C)Cl
CC(Cl)(Cl)Cl
C1CCCNC1
C(=O)([2H])O[2H]
c1cccnc1
F[Sb](F)(F)(F)F
Cc1ccccc1
N([2H])(c1c([2H])c([2H])c(c(c1[2H])[2H])[2H])[2H]
CCCCCCCCCCCCCCCC
C1=CCC=CC1
[O-][N+](=O)c1ccccc1
c1ccc2c(c1)nccc2
C[C@]12CC[C@H](CC1)C(O2)(C)C
C1CCCC=CCC1
CCOCCOCCOCC
CSC
COC(=O)OC
Fc1c(F)ccc(c1F)F
CC(=O)N(C)C
C1CC=CC1
C[As]([As](C)C)C
CCOCC
COc1ccccc1
c1ccncn1
Fc1ccccc1
ClCC(Cl)Cl
CCCCC(=O)C
O
CCCCCCC
CCC(C)(C)C
CC(Cl)(C)C
OS(=O)(=O)O
O[Cl](=O)(=O)=O
ClCCCl
Cc1cccc(c1)C
FC(C(C(F)(F)F)(F)F)(C(C(C(F)(F)F)(F)F)(F)F)F
FCC(F)(F)O
OC=O
Cc1ncc(nc1)C
c1([2H])c([2H])nc(c(c1[2H])[2H])[2H]
COCOC
CCC(=O)C
CCCCCCCCCN
Cc1cncc(c1)C

CCCCCCCCCC
NCC=C
Cc1cccc(c1N)C
CC#N
CC(COC(=O)C)C
C[C@@H]1CC[C@H](CC1)C
CC(=C)C#N
CC([N+](=O)[O-])C
Ic1ccccc1
CC(N)C
c1ccc[nH]1
O([2H])C([2H])([2H])[2H]
c1ccc(cc1)/C=C\c1ccccc1
COC(OC)OC
ClC(Cl)(Cl)Cl
CCC#N
C1CCCCC1
Cc1cnccc1C
COCCOC
CCC1CCCCC1
CC(=O)c1ccccc1
CCCCCCCCCCC
COC=O
CCOC(=O)C
COCCOCCOCCOCCOC
CC(OC(C)C)C
N#CC(C)(C)C
NN
Cc1cc(C)cc(c1)C
O=C1CCCN1C
COC(=O)C(=C)C
CCOC(=O)CC
C#Cc1ccccc1
CCCCCCC(=O)O
CC=C(C)C
CCCCC(=O)O
CNC=O
Cc1ccccn1
CCCCCCCCO
CN(P(=O)(N(C)C)N(C)C)C
CN(CCN(C)C)C
NCCCN
CC1CCCCC1
CCCCCN
CI
CC(c1ccccc1)C
ClC=C(Cl)Cl
CCCC=C
C1CCCOC1

[ClH]1C[ClH]C1
c1ccccc1
CCCCN
C1CCCS1
ClC(=O)c1ccccc1
COC(=O)C
CC[Zn]CC
N#CCc1ccccc1
Cc1cccc2c1cccc2
[O-][N+](=O)OC
C=Cc1ccccc1
[O-][N+](=O)CC
CCCCCOC(=O)C
CCc1ccccc1
c1ccnnc1
CCCCOC(=O)C
FC(c1cccc(c1)C(F)(F)F)(F)F
C[Si]([Si](C)(C)C)(C)C
S=C=S
C[Si](C)(C)C
CN1CCCC1
O=C1CCCO1
BrCBr
Clc1ccccc1
CCc1cc(CC)cc(c1)CC
CCCCCC(=O)O
OCc1ccco1
CC1COC(=O)O1
OCC(C)C
Fc1cc(F)c(cc1F)F
CCCOC(=O)C
N#Cc1c(F)c(F)c(c(c1F)F)F
C1CCC2C(C1)CCCC2
CC(=O)CC(O)(C)C
C([2H])(Br)(Br)Br
COC(C)(C)C
C([2H])(c1c([2H])c([2H])c(c(c1[2H])[2H])C([2H])([2H])[2H])([2H])[2H]
CC(C1CCCCC1)C
C1CNCCO1
COCCOCCOC
O=Cc1ccccc1
CC(=O)C(C)C
FC(S(=O)(=O)O)(F)F
C1CCC=CC1
CC(CC(C)(C)C)C
CC(O)C
OCCO
CCOC(C)(C)C
CNCC[N](CCN(C)C)(C)C

COC(=O)c1ccccc1
C1CCC(CC1)NC1CCCCC1
Cc1cc(C)nc(c1)C
CO
CC#CC
Fc1cccc(c1)F
Brc1ccccc1
Fc1cc(F)c(c(c1F)F)F
NCCN
CCCC(O)C
C([2H])(S(=O)C([2H])([2H])[2H])([2H])[2H]
NCCCCCN
CCCCOCCCC
C[Si](N[Si](C)(C)C)(C)C
Fc1cc(F)c(c(c1)F)Br
C1=CC=CC=CC=C1
Cc1ccccc1C
CN(C(=O)N(C)C)C
C1CCN1
C1OCCO1
C[Si](Cl)(C)C
C1CCCO1
ClC(C(Cl)(Cl)Cl)Cl
C1CCc2c(C1)cccc2
CC(=O)OC(=O)C
c1([2H])c([2H])nc(c(c1C)[2H])[2H]
OCCOCCOCCOCCO
C1CCCN1
CCCC(C)C
c1cccs1
COC(OC)(C)C
CC(OC(=O)C)C
COCCOCCO
Nc1ccccc1Cl
CCCCCCCCC(=O)O
Cn1cncc1
OCCOCC
c1([2H])c(c([2H])c(c(c1C([2H])([2H])[2H])[2H])[2H])C([2H])([2H])[2H]
CC(=O)O
Cc1cccc(c1)O
NCCNCCN
CNN
CC(=O)C
COCCO
CCCO
CCCN
CCCCCCCC
C([2H])(Cl)(Cl)Cl
CCCOCCC

CCC(C)C
C([2H])(C(=O)C([2H])([2H])[2H])([2H])[2H]
CCCCCCCCCCCCCC
CCCCC(CO)CC
ClC(Cl)Cl
CCC(=O)CC
C1CCO1
OCCC(C)C
CC(=O)OC=C
CCCCCCCC(=O)O
CCCCCCCCC
CC(=O)C(C)(C)C
CCCCCCCCCC(=O)O
CS(=O)C
CC1CCCC1
c1([2H])c([2H])c(c(c(c1[2H])[2H])C([2H])([2H])[2H])C([2H])([2H])[2H]
CCC(C(=O)O)(C)C
CCB(CC)CC
CCCCCCCC(=O)C
C1CCCCOC1
CCCCCCCCN
[2H]C(Cl)(Cl)Cl
O=N(=O)c1ccccc1
[2H]c1c([2H])c([2H])c(c(c1[2H])[2H])[2H]
CN(=O)=O
O[C@@H](C(F)(F)F)c1ccccc1
[2H]C(C(=O)C([2H])([2H])[2H])([2H])[2H]
N1CCNCC1
CCBr
COc1cccc(c1)C
COc1ccc(cc1)C
[BrH4]CC[BrH4]
[BrH4]CCCC[BrH4]
[2H]O[2H]
[2H]OC(=O)C(F)(F)F
ClCCCCCl
FCCCCF
CCc1ccncc1
Clc1cccnc1
OC(O)O
NCCO
OCCOCCOCC
N#C/C=C/C=C/C#N
CC(CCOC(=O)C)C
OCC#N
CC[C@@H](N)C
O=C1CCCN1
CCc1ccccn1
C[C@@H]1CCC[C@@H]2[C@@H]1CCC[C@@H]2C

F[C@@H]1CCC[C@H]2[C@H]1CCC[C@@H]2F
Brc1cccc2c1cccc2
Clc1cccc2c1cccc2
CCOC(=O)O
Cc1ccccc1Cl
OOC(C)(C)C
OOC(c1ccccc1)(C)C
[2H]C(Cl)(Cl)[2H]
Br/C=C\\Br
CCOC(=O)C(=O)OCC
BrCCBr
C[C@@H]1CCCCC1=O
C[C@@H]1CCCC(=O)C1
CC1CCC(=O)CC1
Clc1ccc(c(c1)Cl)Cl
N1CC21NC2
OCCCCO
O=C1N(C)CCCN1C
[2H]C(S(=O)C([2H])([2H])[2H])([2H])[2H]
OCC(F)F
CN1CCOCC1
CCNC(=O)C
O1CC1
CN(c1ccccc1)C
OCCc1ccccc1
NCc1ccccc1
Cc1c(C)cccc1C
Cc1ccc(c(c1)C)C
BrBr
CCCBr
CCCCl
O=Cc1ccncc1
[2H]OC([2H])([2H])[2H]
CC(=C)C(=C)C
O=Cc1ccco1
[2H]C(C#N)([2H])[2H]
ClC(=C(Cl)Cl)Cl
CCOCCOCC
COc1ccc(cc1)C=O
FC(c1ccccc1)(F)F
[2H]c1c([2H])c([2H])c(c(c1C([2H])([2H])[2H])[2H])[2H]
C[C@H](c1ccccc1)O
CC[C@@H](Cl)C
CCCCCl
ClC/C=C/CCl
C/C(=C/C(=O)C)/O
ClCBr
Fc1c(F)c(F)c(c(c1F)I)F
Cc1cccs1

Cc1cscn1
N#CC1CCCCC1
N#CN1CCCC1
O=C1CC=C(O1)C
O=C1CCCCCC1
OC1CCCC1
N#CCCCCCCC#N
CCOC(=O)CC(=O)OCC
Cc1ccno1
ClC(Br)Cl
C1CCC=CC=CC1
C1CC=CCCC=C1
NC1CCCCCC1
NC1CCCCC1
NC1CCCC1
CCCCNCCCC
CC(NC(C)C)C
CCN(C)C
CCCNCCC
CN(C1CCCCC1)C
CC(CC(N)(C)C)(C)C
OC[C@H](O)C
[2H]C(N(=O)=O)([2H])[2H]
CCCCOCCO
CCOC(=O)N=C=S
CCN(=O)=O
C[C@@H]1CS1
CCOC(=O)OCC
Fc1ccc(cc1)F
ClCCSCCCl
ClCCSCC
ClCCOCC
CCCCCCl
ClCCOCCCl
ClCCCCCCl
O=Cc1c(F)c(F)c(c(c1F)F)F
OO[C@H]1COCCO1
CCCC
COc1cccc(c1)OC
CO[C@@H]1CO[C@H]2[C@@H]1OC[C@@H]2OC
CNc1ccccc1
BrCCCCBr
Cc1ccc(cc1)Cl
O=C1CCCC=C1
[2H]C1([2H])OC(C(C1([2H])[2H])([2H])[2H])([2H])[2H]
C[C@H]1CCC(=O)O1
O=S1CCCC1
CC[C@@H](CO)C
CC(=C)c1ccccc1

CCCCC=O
CCCCCC=O
Clc1ccccc1Br
N(Cc1ccccc1)Cc1ccccc1
CCO[C@H](O)C
C[C@H]1O[C@@H]1C
[2H]C(C(Cl)(Cl)[2H])(Cl)Cl
O1CCOCCOCCOCC1
OB1[C@H]2CCC[C@@H]1CCC2
CC(=O)OC(COC(=O)C)COC(=O)C
C/C=C/C
N#CCCCCCCCCCCC#N
CCOP(=O)(OCC)OCC
CCCCC#N
N#CC1CC1
BrCCCCCCCCBr
CC(=O)N1CCOCC1
CC(C(=O)O)C
CCC(CC)O
OC1CCCCCCC1
N[C@@H]1CCCC[C@H]1Cl
Oc1ccccc1Cl
CC1=CC(=O)CC(C1)(C)C
O=N(=O)c1ccccc1C
Cc1ncccc1C
Cc1cccc(n1)C
Cc1ccccc1Br
Cc1cccc(c1)Br

References

[1] Groom C, Bruno I, Lightfoot M, Ward S. The Cambridge Structural Database. Acta Crystallographica Section B Structural Science, Crystal Engineering and Materials. 2016 04;72:171-9.

[2] Devogelaer JJ, Meekes H, Vlieg E, de Gelder R. Co-crystals in the Cambridge Structural Database: a network approach. Acta Cryst. 2019;B75:371-83.

[3] de Vries TE, Vlieg E, de Gelder R. Unravelling the structure of the CSD cocrystal network using a fast near-optimal bipartisation algorithm for large networks. CrystEngComm. 2024;26:192-202. Available from: http://dx.doi.org/10.1039/D3CE00978E.

[4] Peng S, Yang H, Yamamoto A. BERT4FCA: A method for bipartite link prediction using formal concept analysis and BERT. PLOS ONE. 2024 06;19(6):1-23. Available from: https://doi.org/10.1371/journal.pone.0304858.

[5] Zhang Y, Phillips C, Rogers G, Baker E, Chesler E, Langston M. On finding bicliques in bipartite graphs: A novel algorithm and its application to the integration of diverse biological data types. BMC bioinformatics. 2014 04;15:110.

[6] de Vries TE, van Eert E, Weevers L, Tinnemans P, Vlieg E, Meekes H, et al. Optimizing Link Prediction for the CSD Cocrystal Network: A Demonstration Using Praziquantel. Crystal Growth & Design. 2024;24(12):5200-10. Available from: https://doi.org/10.1021/acs.cgd.4c00438.
